# Modern synthetic pathways towards eribulin and its subunits

**DOI:** 10.3762/bjoc.22.37

**Published:** 2026-03-19

**Authors:** Sebastian Dominik Graf

**Affiliations:** 1 Gertraud-Kaltenecker Straße 24, D-93049 Regensburg, Germany

**Keywords:** breast cancer, drug manufacturing, eribulin, Halaven, total synthesis

## Abstract

Eribulin is a synthetic analog of halichondrin B, a natural product derived from marine sponges, and has gained significant importance in oncology (as its commercial mesylate salt, Halaven) due to its unique mechanism of action as a microtubule dynamics inhibitor. It is primarily used in the treatment of metastatic breast cancer and liposarcoma, offering a new therapeutic option for patients with advanced disease. To meet the increasing clinical demand, the research on new synthetic approaches is rigorously ongoing. Recent procedures mainly focus on more efficient and scalable techniques for the assembly of the 4 key fragments of eribulin. But also new pathways for the total synthesis have emerged in the last decade. In this review the latest advancements towards the construction of eribulin are summarized.

## Introduction

**Figure 1:**
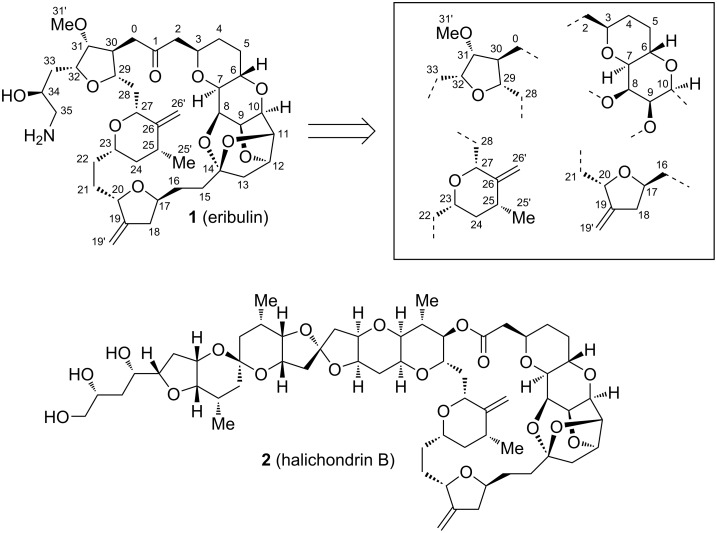
Eribulin with common synthetic precursor fragments and halichondrin B.

Eribulin (**1**) is a truncated derivative of halichondrin B (**2**), a complex natural product originally isolated from the marine sponge *Halichondria okadai* (Figure 1) [1–5]. Already within their isolation study on halichondrin B, in 1986, Hirata and Uemura showed its promising activity against murine cancer cells [6], which led to a great interest in the pharmaceutical society [7–20]. Only 6 years later, Kishi and co-workers first described the total synthesis of the marine natural product [19] and shortly thereafter, also its simplified structure, **1**, was assembled and showed similar anticancer behavior [19,21]. Since 2010, the mesylate salt of **1** is approved by the U.S. Food and Drug Administration (FDA) for the treatment of patients with locally advanced breast or metastatic cancer and has evolved to a commonly used agent for this type of cancer in nowaday’s medicine (commercial name: Halaven) [22–30]. Therefore, the discovery of **1** also marked a significant milestone in the field of medicinal chemistry, as it exemplifies the successful translation of marine natural products into effective therapeutic agents.

The clinical importance of **1** primarily stems from its efficacy in treating aggressive and refractory cancers, notably metastatic breast cancer and liposarcoma [31–35]. Historically, treatment options for advanced breast cancer have been limited, especially after patients have progressed on initial therapies such as anthracyclines and taxanes [36–40]. **1** has demonstrated a significant survival benefit in this setting. Similarly, in liposarcoma, a rare but challenging soft tissue sarcoma, **1** has shown promise in prolonging overall survival and improving quality of life. The key feature that underpins **1**'s clinical importance is its unique mechanism of action [41–50]. Unlike other microtubule inhibitors such as taxanes and vinca alkaloids, **1** binds to a specific site on tubulin, inhibiting microtubule growth without affecting its disassembly. This results in the suppression of mitotic spindle formation, leading to cell cycle arrest at the G2/M phase and subsequent induction of apoptosis. Its distinctive mode of action not only enhances its therapeutic efficacy but also helps in overcoming resistance mechanisms that limit the effectiveness of other microtubule-targeting agents.

Research continues to explore additional applications of **1** in various cancer types, including non-small cell lung cancer, ovarian cancer, and other soft tissue sarcomas. Moreover, ongoing studies aim to optimize combination therapies involving **1** with targeted agents, immunotherapies, and other chemotherapeutics to enhance its efficacy and reduce adverse effects [51–65].

**Scheme 1:**
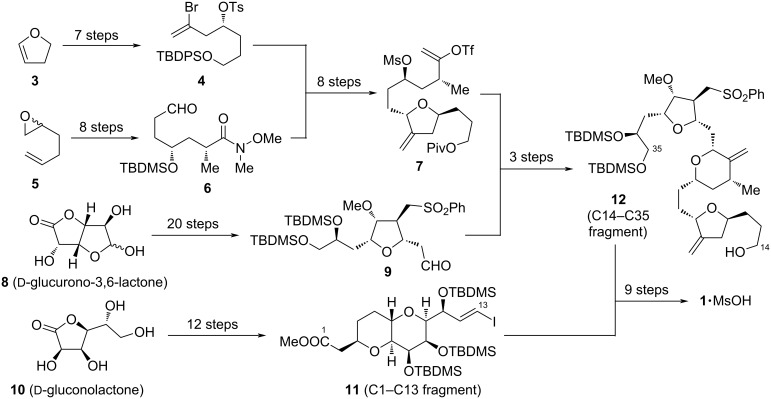
Overview of the industrial process pathway for the large-scale production of the mesylate salt of **1** by Eisai.

Given the challenging structure of **1**, its rapid development from first total synthesis to large scale production also highlights advances in the realm of synthetic chemistry. This progress was indispensable to ensure today`s broad accessibility, since only smallest quantities would be obtainable through isolation form its natural source (12.5 mg from 600 kg *Halichondria okadai*) [6]. The commercial manufacture of **1** is carried out by the company Eisai and involves multiple linear and convergent synthesis paths (Scheme 1, 67 steps in total with the longest linear sequence of 32 steps from **8**→**9**→**12**→**1**), which aim towards the merger of C1–C13 fragment **11** with C14–C35 fragment **12** [66–68]. Despite these great advances, still, the research on improving synthetic efficiency, reducing production costs, omitting toxic chemicals, as well as on new pathways towards **1**`s 4 heterocyclic precursor fragments is rigorously ongoing [69–71]. In 2016, Bauer already reported on the current state of research, focusing on contributions from Kishi and co-workers and the Eisai process [71]. However, due to the great demand of **1**, this research field continues to grow. In this context, the following review should summarize and explain modern approaches towards the key fragments and total synthetic strategies for **1** in recent years.

## Review

**Scheme 2:**
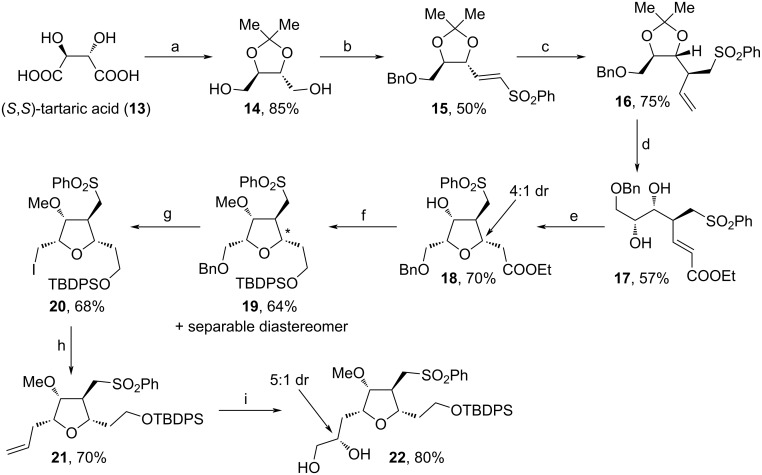
Synthesis of **22**. (a) i. 2,2-dimethoxypropane, *p*-TsOH, MeOH, 65 °C; ii. NaBH_4_, MeOH, rt; (b) i. NaH, BnBr, THF, rt; ii. iodobenzoic acid, MeCN, 80 °C; iii. PhOOSCH_2_PO(OEt)_2_, NaH, THF, 0 °C; (c) vinylmagnesium bromide, CuI, THF, −78 °C; (d) i. ethyl acrylate, Grubbs cat 2nd generation, toluene, 110 °C; ii. *p*-TsOH, H_2_O, THF, 60 °C; (e) NaH, THF, 0 °C; (f) i. LiAlH_4_, THF, 0 °C to rt; ii. TBDPSCl, imidazole, DCM, 0 °C to rt; iii. MeI, Ag_2_O, DMF, 0 °C to rt; (g) i. Pd/C, H_2_, EtOAc; ii. I_2_, PPh_3_, imidazole, DCM, 0 °C to rt; (h) vinylmagnesium bromide, CuI, HMPA, THF, −30 °C; (i) OsO_4_, (DHQ)_2_PYR, 0 °C; (DHQ)_2_PYR: hydroquinine 2,5-diphenyl-4,6-pyrimidinediyl diether.

**Scheme 3:**
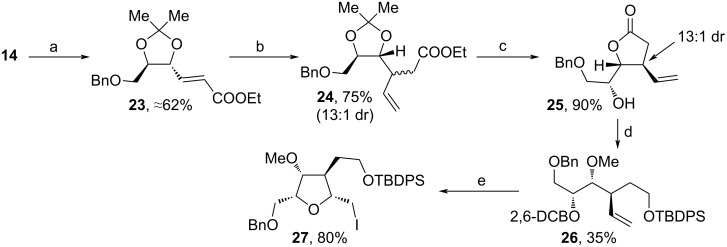
Synthesis of **27**. (a) i. NaH, BnBr, THF, rt; ii. iodobenzoic acid, MeCN, 80 °C; iii. (EtO)_2_POCH_2_COOEt, NaH, THF, 0 °C; (b) vinylmagnesium bromide, CuI, THF, TMSCl, HMPA, −78 °C to rt; (c) 2 M HCl, THF, H_2_O, 65 °C; (d) i. Ag_2_O, 2,6-DCBCl, DMF, TBAI, rt, darkness; ii. LiAlH_4_, THF, 0 °C to rt; iii. TBDPSCl, imidazole, DCM, 0 °C to rt; iv. NaH, MeI, DMF, 0 °C; (e) I_2_, MeCN, −20 °C; 2,6-DCBCl: 2,6-dichlorobenzyl chloride.

In 2016, Konda and co-workers reported two approaches for the assembly of the tetrasubstituted tetrahydrofuran unit of **1** (Scheme 2 and Scheme 3) [72]. For the first path, (*S*,*S*)-tartaric acid (**13**) was used as a starting material and was protected as acetonide within the first step to enable the reduction of both acid moieties towards **14** (Scheme 2). Bn-protection, followed by oxidation and olefination yielded sulfone **15**, which was vinylated leading to **16** as a single diastereomer. Further Grubbs metathesis with ethyl acrylate, acidic cleavage of the diol protecting group and addition of NaH induced the oxy-Michael reaction towards **18** in 4:1 dr. Reduction of the ester moiety, subsequent protection with TBDPSCl and methylation of the secondary alcohol furnished **19**. After Bn-deprotection, iodination and addition of vinylmagnesium bromide, **21** was received and dihydroxylated towards **22** in 5:1 dr.

The second path towards **27** commenced with the assembly of **23** from **14** via Bn-protection, following oxidation and Horner–Wadsworth–Emmons (HWE) reaction (Scheme 3). Stereospecific vinylation with a Gilman cuprate and acidic treatment afforded **25** in 13:1 dr. After protection with 2,6-DCBCl, reduction with LiAlH_4_, TBDPS-protection and methylation, **26** was received in 35% yield. Eventually, the addition of I_2_ triggered an iodocyclization towards **27**. While in the first sequence only mg amounts of **22** were received, 1.45 g of **27** were obtainable during the second one from one batch (Scheme 3, step e) showing the scalability of this path. Moreover, besides **22** and **27**, Konda and co-workers also accomplished the stereoselective syntheses of other diastereomers of the target tetrahydrofuran unit starting from **13**.

**Scheme 4:**
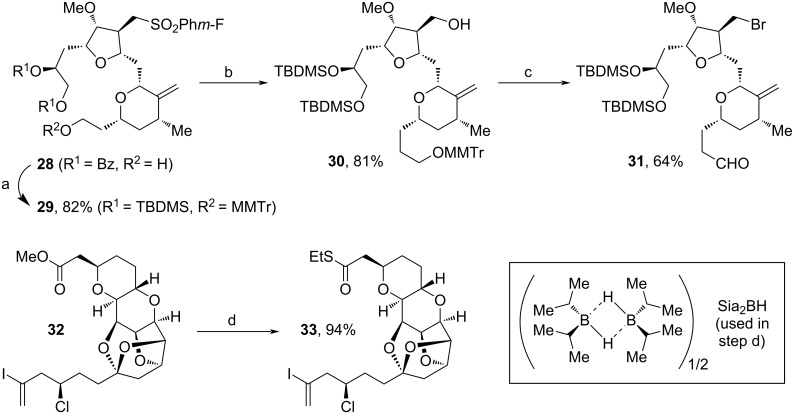
Synthesis of **31** and **33**. (a) i. MMTrCl, iPr_2_NEt, DCM, rt; ii. K_2_CO_3_, MeOH, DCM, rt; iii. TBDMSCl, imidazole; (b) i. *n*-BuLi, THF, −78 °C, Sia_2_BH, −10 °C to rt; ii. H_2_O_2_, 3 M NaOH, 0 °C; (c) i. TsCl, DMAP, NEt_3_, DCM, rt; ii. NaBr, *n*-Bu_4_NBr, acetone, reflux; iii. HFIP, H_2_O, rt; iv. DMP; (d) i. Me_3_SnOH, DCE, 80–85 °C, then 0.1 M HCl; ii. EtSH, DCC, DMAP, DCM, rt; MMTrCl: 4-methoxytriphenylmethyl chloride; Sia_2_BH: disiamylborane; HFIP: 1,1,1,3,3,3-hexafluoroisopropanol.

**Scheme 5:**
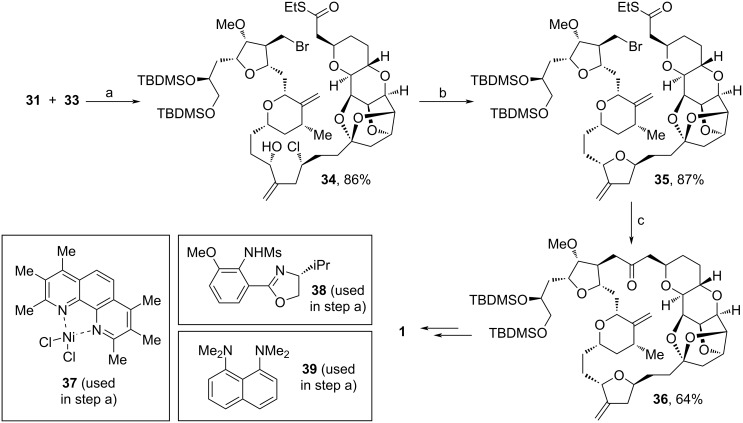
Synthesis of **1**. (a) CrCl_2_, **37**, **38**, **39** (proton sponge), LiCl, Mn, ZrCp_2_Cl_2_, MeCN, EtOAc; (b) SrCO_3_, *t*-BuOH, H_2_O, open air; (c) Pd_2_dba_3_, PCyp_3_, CrCl_3_, NbCpCl_4_, Zn(0), DMI, THF; PCyp_3_: tricyclopentylphosphine; DMI: 1,3-dimethyl-2-imidazolidinone.

The Kishi group has made major contributions in the area of **1** synthesis over the last three decades [69,71]. Also recently, the group established a versatile protocol for the macrocyclization towards precursor **36** (Scheme 4 and Scheme 5) [73]. Herein, fragments **31** and **33** were fused via Nozaki–Hiyama–Kishi (NHK) coupling and Pd-mediated cyclization. Fragment **31** was synthesized from known precursor **28** [74] in 9 steps via MMTr-protection, replacement of the Bn- with TBDMS-protecting groups, hydroxylation of sulfone **29** to alcohol **30**, tosylation, bromide substitution, acidic MMTr-cleavage and DMP-oxidation (Scheme 4, above). For the assembly of **33**, only the hydrolysis of previously reported **32** [75] with Me_3_SnOH and thioesterification using EtSH and DCC were necessary (Scheme 4, below).

Both fragments (**31** and **33**) were fused together via NHK coupling to furnish **34** in 86% yield. The addition of SrCO_3_ proved to be suitable for the cyclization towards **35** and the eventual macrocyclization was achieved via coupling of the alkyl bromide unit with the thioester. Mechanistically, this reaction is enabled by the formation of an intermediate alkylzinc halide, which is produced by single electron transfer using CrCl_3_ and NbCpCl_4_. The last steps towards **1** are known procedures [68]. By comparison to former methods, this technique does not require further desulfonylation after the macrocyclization [19,68].

**Scheme 6:**
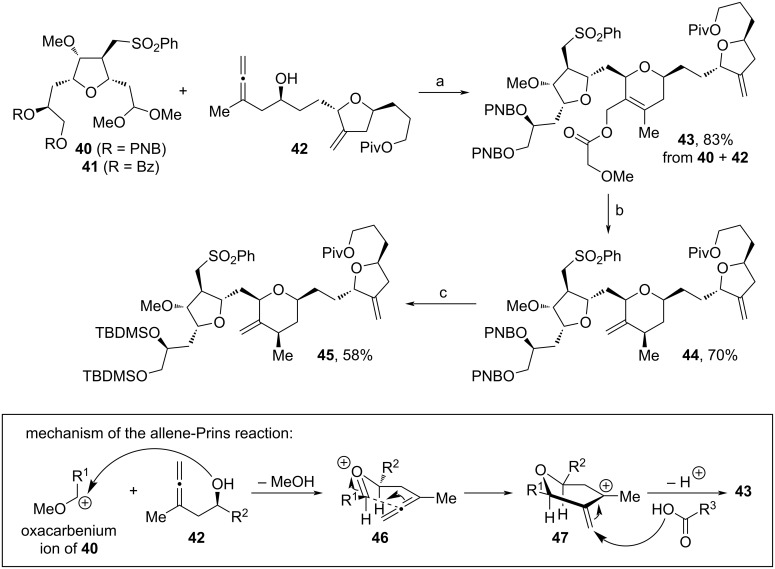
Synthesis of **45**. Above: Reaction conditions: (a) methoxyacetic acid, BF_3_·OEt_2_, DCM, −30 °C; (b) Pd(PPh_3_)_4_, PPh_3_, HCOOH, NEt_3_, THF, 60 °C; (c) i. Mg(OMe)_2_, THF, MeOH, rt; ii. TBDMSCl, imidazole, DMF, rt. Below: Mechanism of the allene-Prins reaction.

Choi and co-workers used a previously reported protocol on stereo- and regioselective allene-Prins reactions [76–77] for the assembly of fragment **45** (Scheme 6) [78]. Here, **40** and **42** served as the substrates for the allene-Prins reaction towards **43**. Notably, the Bz-derivative of **40**, **41**, served as a starting point for a corresponding halichondrin B analog. The stereoselective course of this cyclization is described in Scheme 6 below (R^1^ and R^2^ in equatorial position). From **43**, the methylene unit in **44** was formed by Pd(0)-mediated generation of a π-allyl–palladium intermediate, followed by reductive termination (Tsuji-reduction), alongside the substrate-controlled alignment of the adjacent methyl substituent. Eventual change of the two PNB- with TBDMS-protecting groups yielded **45** in 58%. Notably, by this technique, the central pyran motif was assembled in one step and required only the stereoinformation of **42**`s alcohol unit, while in former works two individual steps and the use of expensive metal catalysts were necessary [66–68,73,79].

**Scheme 7:**
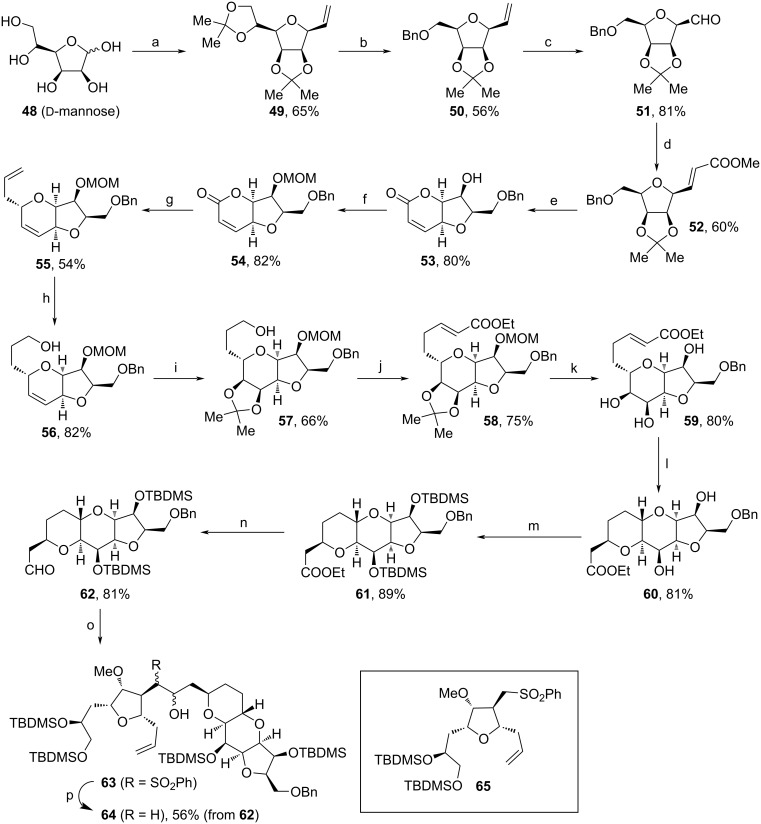
Synthesis of **64**. Reaction conditions: (a) i. acetone, I_2_, rt; ii. vinylmagnesium bromide, THF, −20 °C to rt; iii. TsCl, pyridine, 65 °C; (b) i. AcOH, rt; ii. NaIO_4_, MeOH, 0 °C to rt; iii. NaBH_4_, MeOH, 0 °C to rt; iv. NaH, BnBr, 0 °C to rt; (c) i. O_3_, DCM, −78 °C; ii. SMe_2_; (d) (PhO)_2_POCH_2_COOMe, KHMDS, 18-crown-6, THF, −78 °C; (e) *p*-TsOH, toluene, H_2_O, 100 °C; (f) MOMCl, DIPEA, DCM, 0 °C to rt; (g) i. DIBAL-H, DCM, −78 °C; ii. Ac_2_O, pyridine, DMAP, DCM, rt; iii. allyl-TMS, BF_3_·Et_2_O, DCM, −78 °C; (h) 9-BBN, THF, H_2_O_2_, 0 °C to rt; (i) i. AD-mix β, MsNH_2_, *t*-BuOH, H_2_O, rt; ii. 2,2-dimethoxypropane, (±)-CSA, DCM, rt; (j) i. DMP, DCM, rt; ii. Ph_3_PCH_2_COOEt, toluene, rt; (k) *p*-TsOH, EtOH, reflux; (l) i. DBU, toluene, reflux; ii. TBAF, THF, rt; (m) TBDMSCl, imidazole, DCM, 50 °C; (n) DIBAL-H, toluene, −78 °C; (o) **36**, *n*-BuLi, −78 to −50 °C; (p) i. DMP, DCM, rt; ii. SmI_2_, MeOH, THF, −78 to −50 °C; 18-crown-6: 1,4,7,10,13,16-hexaoxacyclooctadecane, 9-BBN: 9-borabicyclo[3.3.1]nonane, (±)-CSA: camphorsulfonic acid.

In early 2018, Gaddam and co-workers assembled the northern fragment of **1** from ᴅ-mannose (**48**, Scheme 7) [80]. The synthesis commenced with the protection of **48** as acetonide, vinylation and cyclization to **49** by the treatment with TsCl according to a procedure from Liu and co-workers [81]. Regioselective deprotection, oxidative cleavage, reduction with NaBH_4_ and Bn-protection afforded **50**, which was subsequently transformed to aldehyde **51** via ozonolysis and treatment with SMe_2_. Next, HWE reaction and acidic treatment triggered the cyclizations towards enone **53**. Protection of the free alcohol unit enabled the transformation towards **55**, which involved the reduction of lactone to lactole, protection of the alcohol as acetate, BF_3_·Et_2_O-mediated C-allylation of the aldehyde (via oxocarbenium intermediate) and cyclization (oxy-Michael reaction). Hydroboration–oxidation, stereoselective dihydroxylation with AD-mix β and diol-protection yielded acetonide **57**. Another DMP-oxidation, followed by HWE reaction, and deprotection of the diol motifs enabled the cyclization towards **60**. After TBDMS-protection and reduction to the respective aldehyde (**62**), an alkylation with the α-sulfonyl carbanion of **65** (intermediate from the Eisai process [68]) via interrupted Julia olefination was performed to furnish **63**. Eventually, **64** was received by oxidation and SmI_2_-induced desulfonylation. Herein, Gaddam and co-workers enable the synthesis of **1** by the merger of northern fragment **64** with a potential southern fragment (ongoing research by the same group) and thereby provide an alternative approach to the current one by Eisai [66].

**Scheme 8:**
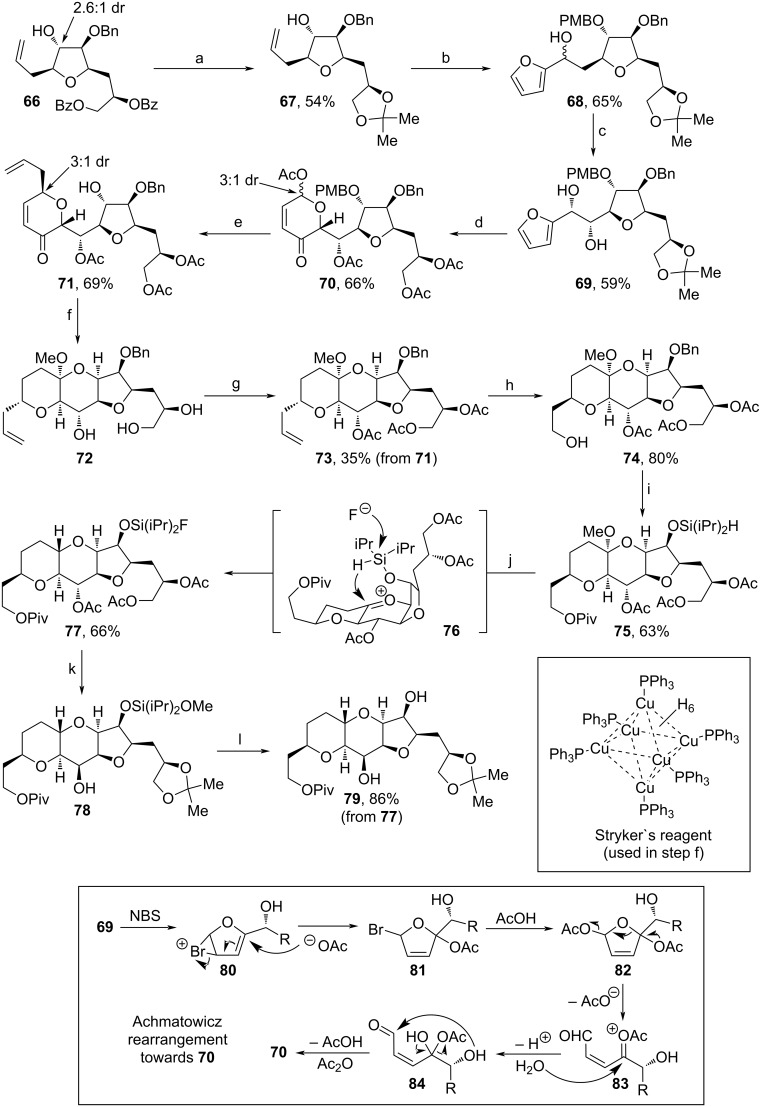
Synthesis of **79**. Above: Reaction conditions: (a) i. K_2_CO_3_, MeOH, 60 °C; ii. 2,2-dimethoxypropane, H_2_SO_4_ (aq), acetone, rt; (b) i. PMBCl, *t*-BuOK, TBAI, DMF, THF, rt; ii. OsO_4_, NaIO_4_, 2,6-lutidine, 1,4-dioxane, H_2_O, −20 °C; iii. *N*-BuLi, furan, THF, 0 °C; (c) i. (PhO)_3_PMeI, dimethylacetamide, rt; ii. AD-mix-α, *t*-BuOH, H_2_O, rt; (d) i. AcOH, H_2_O, rt; ii. NBS, NaHCO_3_, NaOAc, THF, H_2_O, 0 °C; iii. acetic anhydride, pyridine, DCM, 0 °C; (e) i. allyl-TMS, BF_3_·OEt_2_, MeCN, −10 °C; ii. DDQ, DCM, H_2_O; (f) i. DMP, NaHCO_3_, DCM, rt; ii. NaBH_4_, DCM, MeOH, −78 °C; iii. Stryker’s reagent, toluene, rt; iv. (±)-CSA, MeOH, 60 °C; (g) acetic anhydride, DMAP, DCM, rt; (h) i. OsO_4_, NaIO_4_, 2,6-lutidine, 1,4-dioxane, rt; ii. ʟ-proline, MeOH, −10 °C; iii. NaBH_4_, MeOH, 0 °C; (i) i. PivCl, pyridine, DMAP, DCM, rt; ii. H_2_, Pd/C, MeOH, EtOAc, rt; iii. (iPr)_2_SiHCl, imidazole, DMAP, DCM, rt; (j) BF_3_·OEt_2_, DCM, −20 to −10 °C; (k) i. Mg(OMe)_2_, MeOH, rt; ii. 2,2-dimethoxypropane, pyridinium *p*-toluenesulfonate, acetone, rt; iii. DMP, NaHCO_3_, DCM, rt; iv. NaBH_4_, MeOH, 0 °C; (l) TBAF, THF, rt. Below: Mechanism of the Achmatowicz rearrangement during step (d).

Shortly thereafter, Kim and co-workers made use of **66**, a byproduct formed during the multigram synthesis of Halaven, to assemble **79** (Scheme 8) [82]. **66** was received within a filtrate during this process and isolated as a diastereomeric mixture of 2.6:1. At first, the benzyl moieties of **66** were cleaved off and the diol was reprotected as acetonide to afford **67**. The remaining secondary alcohol was PMB-protected and the olefin was oxidatively cleaved, before addition of a lithiated furanyl unit took place. The so-obtained 1:1 diastereomeric mixture of **68** was treated with (PhO)_3_PMeI in dimethylacetamide and formed an intermediate *trans*-olefin, which subsequently underwent an asymmetric Sharpless dihydroxylation. The acetonide was cleaved with AcOH and the addition of NBS under basic conditions triggered an Achmatowicz rearrangement (shown in Scheme 8, below) to assemble a hydropyranone ring [83]. Eventual acetylation of the free alcohol units afforded **70** in 3:1 dr. C-Glycolysation with allyl-TMS and oxidative cleavage of the PMB moiety yielded **71**. The stereochemistry of the secondary alcohol unit of **71** was changed via oxidation and stereospecific reduction, then 1,4-reduction of the enone and treatment with camphorsulfonic acid led to fully cyclized intermediate **72** as a single isomer. During this process the acetate groups were cleaved off and had to be reinstalled using acetic anhydride. Oxidative cleavage of olefin **73**, followed by treatment with ʟ-proline led to an intermediate β-aldehyde with inverted stereochemistry at the β-C (4:1 dr). Subsequent reduction with NaBH_4_ yielded alcohol **74**. Piv-protection of the primary alcohol, reductive deprotection of the Bn-moiety and reprotection with (iPr)_2_SiHCl afforded **75**. The addition of BF_3_·OEt_2_ at low temperatures induced the fluorination of the silane protecting group and a follow-up intramolecular hydride transfer (via **76**). Afterwards, the introduced fluoride was substituted with Mg(OMe)_2_ in MeOH and the stereochemistry of the central protected alcohol unit was inverted within a sequence involving deprotection of all acetates, protection of the 1,2-diol as an acetonide, oxidation of the central alcohol with DMP and stereospecific reduction, which yielded alcohol **78**. Finally, the silyl protecting group was cleaved with TBAF leading to **79**. Although the synthesis of **79** through this method is quite laborious, especially with regard to all necessary adjustments of stereocenters, the idea of recycling byproducts of Halaven production clearly shows the advancements in this process. Therefore, this route should not be valued solely for its total yield, but rather as a starting point for improving atom economy.

**Scheme 9:**
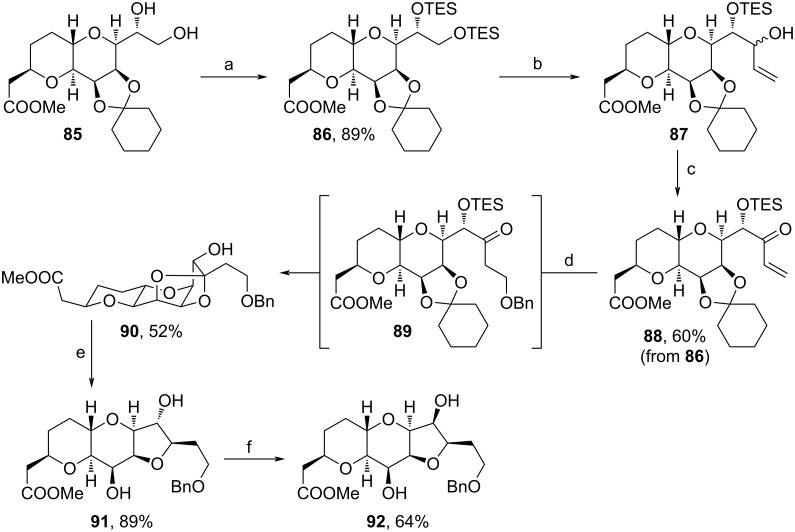
Synthesis of **92**. Reaction conditions: (a) TESCl, imidazole, DCM, 0 °C to rt; (b) i. oxalyl chloride, DMSO, NEt_3_, DCM, −78 °C; ii. vinylmagnesium bromide, THF, −70 °C; (c) DMP, NaHCO_3_, DCM, rt; (d) BnOH, *p*-TsOH·H_2_O, toluene, 70–75 °C; (e) BF_3_·OEt_2_, Et_3_SiH, DCM, 0 °C to rt; (f) i. DMP, NaHCO_3_, DCM, 0 °C; ii. Li(*t*-BuO)_3_AlH, THF, 0−10 °C.

The same group also reported on a metal-free synthesis of **92** starting from an intermediate occuring during the current large-scale production of Halaven (Scheme 9) [84]. Initially, the diol motif of **85** was protected with TESCl, then regioselective oxidation of the primary TES ether and addition of vinyl grignard led to **87**. Another oxidation afforded α,β-unsaturated carbonyl **88** and acidic treatment with BnOH triggered the oxa-Michael reaction and transketalization towards tetracyclic **90**. Following Kishi reduction [85], epimerization of the secondary alcohol was effected via DMP-oxidation, and stereospecific reduction with Li(*t*-BuO)_3_AlH yielded **92**. In comparison to the current pathway from **85** to **92**, which involves the use of heavy metals adding considerable amounts of cost [19,66], this technique stands out for the employment of cheaper and more eco-friendly reagents.

**Scheme 10:**
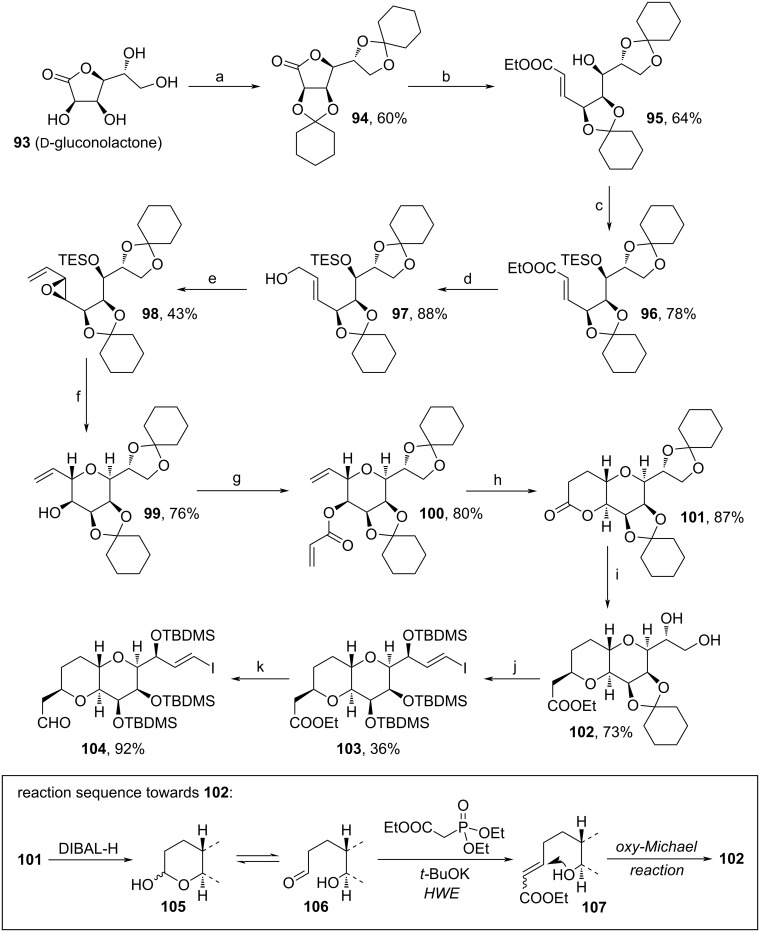
Synthesis of **104**. Above: Reaction conditions: (a) cyclohexanone, *p*-TsOH, toluene, 110 °C, crystallization. (b) i. DIBAL-H, THF/toluene, −10 °C; ii. Ph_3_PCHCO_2_Et, benzoic acid, DCM, 50 °C; (c) i. TESOTf, 2,6-lutidine, DCM, 0 °C; ii. 5 mol % P*n*-Bu_3_ (5 mol %), THF, 50 °C; (d) DIBAL-H, DCM, 20 °C; (e) i. (−)-DET, Ti(OiPr)_4_, DCM, 0 °C; ii. DMP, DCM, 0 °C; iii. MePPh_3_Br, NaHMDS, THF, 0 °C; (f) i. TBAF, THF, 0 °C; ii. pyridinium *p*-toluenesulfonate, DCM, 0 °C; (g) acrylic acid, DIC, DMAP, DCM, rt; (h) i. Grubbs cat 2nd generation, toluene, reflux; ii. Pd/C, H_2_, EtOAc; (i) i. DIBAL-H, THF/toluene, 10 °C ; ii. triethyl phosphonoacetate, *t*-BuOK, THF, 60 °C; iii. AcOH, 40 °C; (j) i. NaIO_4_, EtOAc, 15 °C; ii. CrCl_2_, NiCl_2_, 1-bromo-2-trimethylsilylethene, DMSO, MeCN, 30 °C; iii. AcOH, H_2_O, 95 °C, crystallization; iv. TBDMSOTf, 2,6-lutidine, MTBE, 30 °C, crystallization; v. NIS, MeCN, toluene, TBDMSCl, 35 °C; (k) DIBAL-H, 2,6-di-*t*-Bu-4-hydroxytoluene, toluene, 65 °C. Below: Reaction sequence towards **102**; DIC: *N,N′*-diisopropylcarbodiimide.

In 2019, Lee and co-workers developed a technique towards the octahydropyrano[3,2-*b*]pyran fragment of **1** from ᴅ-gluconolactone (**93**) (Scheme 10) [86]. After protection of **93**, the lactone unit was reduced with DIBAL-H and the emerging aldehyde motif (equilibrium of lactol to aldehyde and alcohol) was trapped by HWE reaction to obtain **95**. The secondary alcohol unit of **95** was protected and the ester reduced to the respective alcohol, before Sharpless epoxidation, oxidation of the alcohol and subsequent Wittig reaction yielded allylic epoxide **98**. Deprotection of the alcohol motif of **98** enabled the cyclization towards tetrahydropyran **99**. Next, **99** underwent an esterification with acrylic acid, was cyclized via Grubbs metathesis and the remaining double bond was hydrogenated leading to bicyclic core structure of **101**. Notably, during the cyclization also considerable amounts of dimerized product were observed. Again, reduction with DIBAL-H led to the regioselective deprotection of the alcohols and reduction to an intermediate lactol unit, which is in equilibrium with its acyclic aldehyde and alcohol. The aldehyde was trapped by HWE reaction and the so obtained α,β-unsaturated carbonyl was reattacked by the adjacent hydroxy moiety via oxy-Michael reaction to form **102**. This sequence is shown in detail in Scheme 10 below. From here, a sequence involving the oxidation of the unprotected 1,2-diol moiety towards an intermediate aldehyde, Ni(II)/Cr(II)-mediated coupling of 1-bromo-2-trimethylsilylethene, acidic cleavage of the remaining cyclohexylidene ring, TBDMS-protection of the three alcohol units and electrophilic substitution of the silyl moiety to afford vinyl iodide **103** was applied. Eventually, ester **103** was reduced to target aldehyde **104**, which contains the necessary functional group pattern to be used as a building block for the assembly of **1**. By the design of this novel synthetic pathway, Lee and co-workers showed that the commonly used NHK reaction involving dual chromium/nickel catalysis can be circumvented [86]. Here, intramolecular ring opening of epoxide **98** and metathesis led to target **104** with high regio- and stereoselectivity.

**Scheme 11:**
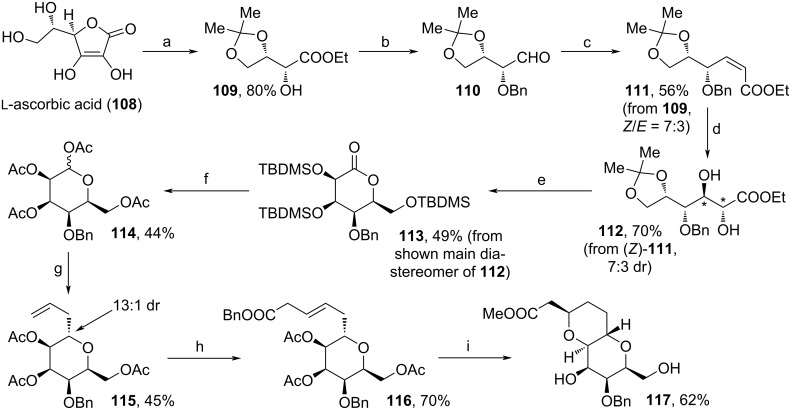
Synthesis of **117**. (a) i. acetone, CuSO_4_, rt; ii. H_2_O_2_, K_2_CO_3_, H_2_O, rt; iii. EtI, MeCN, 70 °C; (b) i. Ag_2_O, BnBr, toluene, rt; ii. LiAlH_4_, THF, rt; iii. (COCl)_2_, NEt_3_, DMSO, DCM, −78 °C; (c) (PhO)_2_POCH_2_COOEt, DBU, THF, −78 °C; (d) K_3_(FeCN)_6_, OsO_4_, K_2_CO_3_, (DHQ)_2_PHAL, *t*-BuOH, H_2_O, 0 °C; (e) i. TFA, H_2_O, MeCN, 70 °C; ii. TBDMSCl, imidazole, DMAP, DMF, 55 °C; (f) i. DIBAL-H, toluene, −78 °C; ii. TBAF, THF, rt; iii. Ac_2_O, NaOAc, 90 °C; (g) BF_3_·OEt_2_, MeCN, 80 °C, allyltrimethylsilane; (h) allyl-COOBn, Grubbs cat 2nd generation, DCM, 40 °C; (i) i. K_2_CO_3_, MeOH, rt; ii. DBU, toluene, 100 °C; (DHQ)_2_PHAL: hydroquinine 1,4-phthalazinediyl diether.

A novel approach towards C1–C10 fragment of **1** was reported by Kathravath and co-workers (Scheme 11) [87]. From ʟ-ascorbic acid (**108**), protection of the diol motif and treatment with H_2_O_2_, then EtI yielded ester **109**. The alcohol motif was protected with BnBr, before the ester unit was completely reduced to the respective alcohol and selectively oxidized to aldehyde **110**. After HWE reaction, the dihydroxylation of (*Z*)-**111** furnished diol **112** in 7:3 dr*.* The shown major diastereomer of **112** was cyclized towards **113** upon acidic treatment and TBDMS-protection. Reduction, cleavage of the silyl-protecting groups and acetylation led to tetrahydropyran **114**. Lewis acid-catalyzed C-allylation, cross metathesis, basic cleavage of the acetate motifs and transesterification enabled the DBU-induced isomerization of **116**’s double bond and following oxy-Michael reaction to the target compound **117**. In total, **117** was obtained in a yield below 1% via the 17-step sequence, mainly due to mediocre yields (partially due to unselective reactions) in steps c→g.

**Scheme 12:**
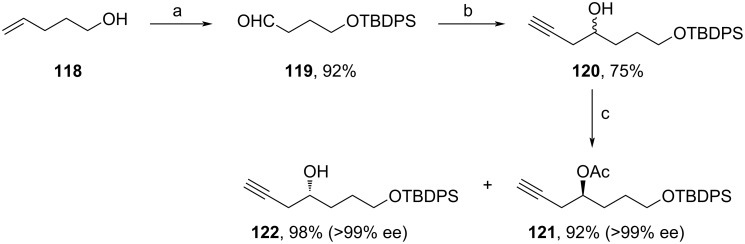
Synthesis of **121**. Reaction conditions: (a) i. TBDPSCl, imidazole, DMF, rt; ii. O_3_, DCM, −78 °C; iii. PPh_3_, rt; (b) propargyl bromide, Zn, NH_4_Cl, THF, −15 °C; (c) enzyme, vinyl acetate, MTBE, continuous flow reactor, rt; enzyme: *Amano lipase* produced by *Pseudomonas fluorescens*.

In the following year, Krishna and co-workers used an enzymatic transformation for the continuous flow production of acetate **121** (C14–C19‘ fragment), which is a starting material needed in large quantities for the total synthesis of **1** (Scheme 12) [88]. Herein, pent-4-en-1-ol (**118**) was protected with TBDPSCl, before ozonolysis, followed by reduction with PPh_3_ yielded aldehyde **119**. The addition of propargyl bromide led to racemic **120**. By the use of a flow setup involving a column packed with *Amano lipase*, an enzyme from the bacterium *Pseudomonas fluorescens*, a kinetic resolution of **120** was performed leading to the continuous production of acetate **121** and free alcohol **122**. Here, **121** bears the right configuration needed for Halaven synthesis, but the authors also showed that **122** was easily converted to **121** via Mitsunobu inversion. Although **121** only represents a small building block for the total assembly of **1**, this method especially stands out for its cost-efficiency and the continuous production in bigger scales (100 mg/mL (272 mM) at 0.1 mL/min).

**Scheme 13:**
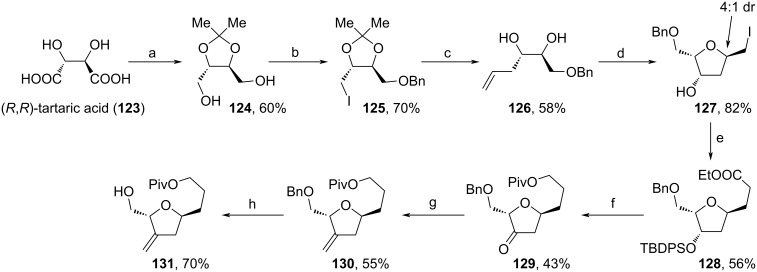
Synthesis of **131**. (a) i. 2,2-dimethoxypropane, *p*-TsOH, MeOH, 60 °C; ii. LiAlH_4_, THF, 0 °C to rt; (b) i. NaH, BnBr, THF, rt; ii. PPh_3_, imidazole, I_2_, THF, 50–60 °C; (c) i. vinylmagnesium bromide, CuI, HMPA, −30 °C; ii. *p*-TsOH, THF, H_2_O, 23 °C; (d) I_2_, NaHCO_3_, Et_2_O, H_2_O, 0 °C; (e) i. TBDPSCl, imidazole, DCM, 0 °C; ii. NaH, diethyl malonate, TBAI, DMF, 120 °C; iii. DMSO, NaCl, 160 °C; (f) i. LiAlH_4_, THF, 0 °C to rt; ii. PivCl, NEt_3_, DCM, 0 °C to rt; iii. TBAF, THF, 0 °C to rt; iv. DMP, DCM, 0−20 °C; (g) PPh_3_MeBr, *n*-BuLi, THF, 0 °C; (h) TiCl_4_, DCM, 0 °C.

In 2021, Mallurwar and co-workers used (*R*,*R*)-tartaric acid (**123**) for the assembly of fragment **131** (Scheme 13) [89]. After diol-protection as acetonide and reduction of the acid motifs to receive diol **124**, one alcohol moiety was Bn-protected, while the other one was iodinated. From **125**, the addition of vinylmagnesium bromide and acidic deprotection furnished diol **126**, which underwent an iodocyclization towards a diastereomeric mixture of **127** (4:1 dr). The shown main diastereomer of **127** could be isolated and underwent TBDPS-protection, which enabled the malonic ester synthesis towards **128**. Reduction with subsequent Piv-protection and TBDPS-deprotection was followed by DMP-oxidation. Finally, the olefination of **129** with PPh_3_MeBr and addition of TiCl_4_ for the cleavage of the Bn-moiety furnished target structure **131**.

**Scheme 14:**
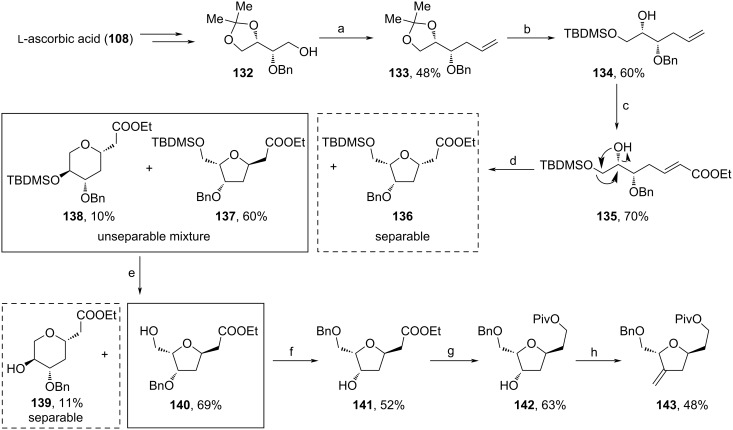
Synthesis of **143**. (a) i. I_2_, PPh_3_, imidazole, DCM; ii. HMPA, CuI, vinylmagnesium bromide, THF, −20 °C; (b) i. *p*-TsOH, MeOH, rt; ii. TBDMSCl, imidazole, DCM; (c) i. OsO_4_, NMO, THF, H_2_O, 0 °C; ii. NaIO_4_, THF, H_2_O, 0 °C; iii. Ph_3_PCHCOOEt, DCM, rt; (d) benzyltrimethylammonium hydroxide, EtOH, 0 °C to rt; (e) *p*-TsOH, MeOH, rt; (f) i. Pd/C, H_2_, EtOAc; ii. Ag_2_O, BnBr, toluene, rt; (g) i. LiAlH_4_, THF, 0 °C; ii. PivCl, NEt_3_, DMAP, DCM, rt; (h) i. DMP, DCM, 0 °C; ii. PPh_3_CH_2_Br, *n*-BuLi, THF, 0 °C to rt.

Within the same work, a similar fragment (**143**) was synthesized using an oxy-Michael reaction as the key step (Scheme 14). Starting material **132** was obtained from natural ʟ-ascorbic acid (**108**) by a sequence involving the protection of diol motif, oxidative cleavage, reduction and regioselective Bn-protection. **132** was further iodinated and reacted with vinylmagnesium bromide to afford **133** in 48% yield. Consecutive acidic deprotection, TBDMS-protection of the secondary alcohol, dihydroxylation, oxidative cleavage and olefination with Ph_3_PCHCOOEt yielded acrylic acid ester **135**. The addition of benzyltrimethylammonium hydroxide induced the oxy-Michael reaction towards an unseparable mixture of **137** and **138**, and **136**. A plausible explanation for the formation of **138** could be a [1,2]-rearrangement of the deprotonated alcohol motif and the OTBDMS-moiety of **137** via an epoxide intermediate (indicated in arrows) and subsequent oxy-Michael reaction. The mixture (**137** + **138**) was treated with *p*-TsOH leading to alcohols **139** and **140**, which were separated via chromatography. From **140**, Bn-deprotection of the primary, followed by Bn-protection of the secondary alcohol unit afforded **141** in 52% yield. Eventually, the ester motif of **141** was reduced to the respective alcohol and Piv-protected, and the secondary alcohol oxidized to an intermediate ketone, then olefinated towards **143**. Although both sequences require multiple applications and removals of protecting groups, they represent attractive pathways towards the C14–C21 fragment of **1** due to the use of inexpensive starting materials.

**Scheme 15:**
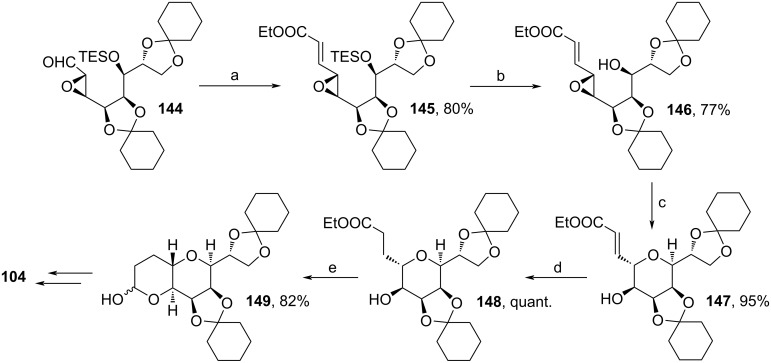
Modified synthesis of **104**. Reaction conditions: (a) (EtO)_2_POCH_2_COOEt, KO*t-*Bu, THF, 15 °C; (b) TBAF, imidazole·HCl, THF, 5 °C to rt; (c) [Rh(CO_2_)Cl]_2_ (0.5 mol %), THF, 60 °C; (d) H_2_, Pd/C, EtOAc, rt; (e) DIBAL-H, toluene, −65 °C.

Kim and co-workers enhanced the procedure from Lee and co-workers by changing few intermediate steps (Scheme 15) [86,90]. From **144**, the authors performed a HWE reaction towards **145** in 80%. Cleavage of the TES-protecting group and Rh(I)-induced cyclization afforded **147** in good yield, which was hydrogenated subsequently. Thereafter, DIBAL-H reduction led to lactol formation towards **149**, which intercepted the former route (Scheme 10, step (i) i.) With these changes, the synthesis was enhanced with regard to scalability of the process. Scaling up the Grubbs metathesis of the first route (Scheme 10, step (h) i.) led to an increasement of dimer yield derived from cross metathesis reactions. Therefore, by the replacement with the Rh(I)-catalyzed cyclization performed herein, this drawback is circumvented and the reactions were performed with kg amounts.

**Scheme 16:**
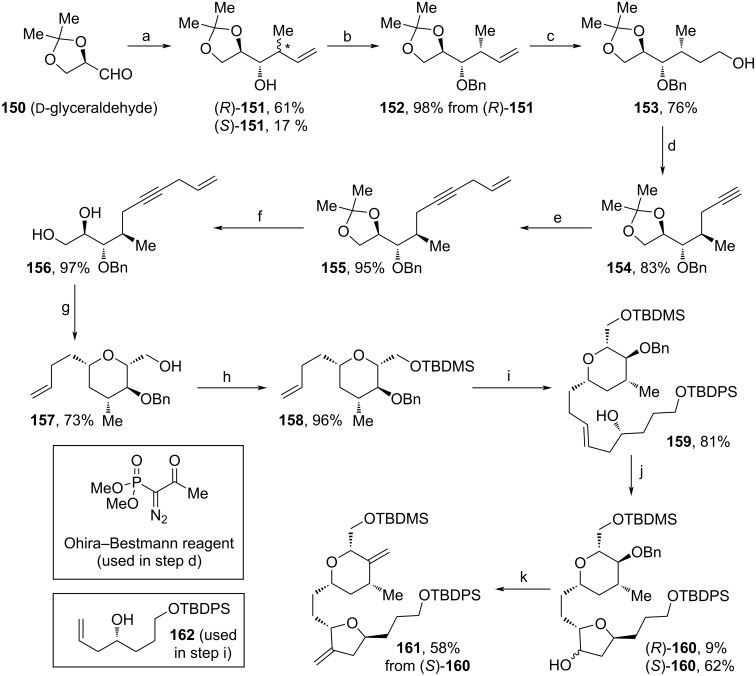
Synthesis of **161**. Reaction conditions: (a) crotyl bromide, Sn, TBAI, NaI, DMF/H_2_O, rt; (b) NaH, BnBr, DMF, rt; (c) 9-BBN, THF, NaOH·H_2_O_2_, EtOH, rt; (d) i. DMP, NaHCO_3_, DCM, rt; ii. Ohira–Bestmann reagent, MeOH, rt; (e) allyl bromide, K_2_CO_3_, CuI, Na_2_CO_3_, DBU, DMF, rt; (f) AcOH (60%), rt; (g) i. Au(PPh_3_)Cl (1 mol %), AgSbF_6_ (1 mol %), DCM, rt; ii. Et_3_SiH, BF_3_·OEt_2_, 0 °C; (h) TBDMSCl, imidazole, DMF, rt; (i) **162**, Grubbs cat 2nd generation, CuI, Et_2_O, 40 °C; (j) i. MsCl, NEt_3_, DCM, 0 °C; ii. AD-mix α, MsNH_2_, *t-*BuOH, H_2_O, 0 °C; (k) i. DDQ, DCM, 45 °C; ii. TFAA, DMSO, DIPEA, DCM, −78 °C; iii. Ph_3_PCH_2_, toluene, 40 °C.

In 2021, Senapati and co-workers developed a route towards key fragment **161** containing the 3-methylenetetrahydrofuran and the 3-methylenetetrahydro-2*H*-pyran motifs of **1** (Scheme 16) [91]. As a cheap and commerically available starting material, ᴅ-glyceraldehyde (**150**) was chosen and allylated with crotyl bromide according to a procedure from Loh and co-workers [92]. The diastereomeric mixture thus obtained was separated via chromatography and (*R*)-**151** was protected using BnBr. After hydroboration–oxidation and DMP-oxidation to the respective aldehyde, Ohira–Bestman reaction was applied to afford **154**. Allylation and acidic deprotection led to diol **156** in excellent yields. Next, Au(I)-catalyzed cyclization and reduction led to tetrahydropyran **157**, which was TBDMS-protected subsequently. The cross metathesis of **158** and **162** (obtained from ʟ-glutamic acid) yielded **159** in 81%. After mesylation of the free alcohol moiety and dihydroxalation using AD-mix α, a diastereomeric mixture of tetrahydropyrans (*R*)-**160** and (*S*)-**160** was obtained and separated for analytical purposes. The Bn-moiety of (*S*)-**160** was cleaved using DDQ and the intermediate diol was further transformed to the respective dicarbonyl via Swern oxidation, and finally olefinated via Wittig reaction. Hence, by the use of one-pot Au(I)-catalyzed cyclization/Kishi reduction for the assembly of the tetrahydropyran and cross metathesis/Sharpless dihydroxylation/etherification for the tetrahydrofuran motifs, this pathway describes a novel and sustainable, but not fundamentally improved alternative in terms of yield towards the structure of **161** (7.2% yield over 14 steps).

**Scheme 17:**
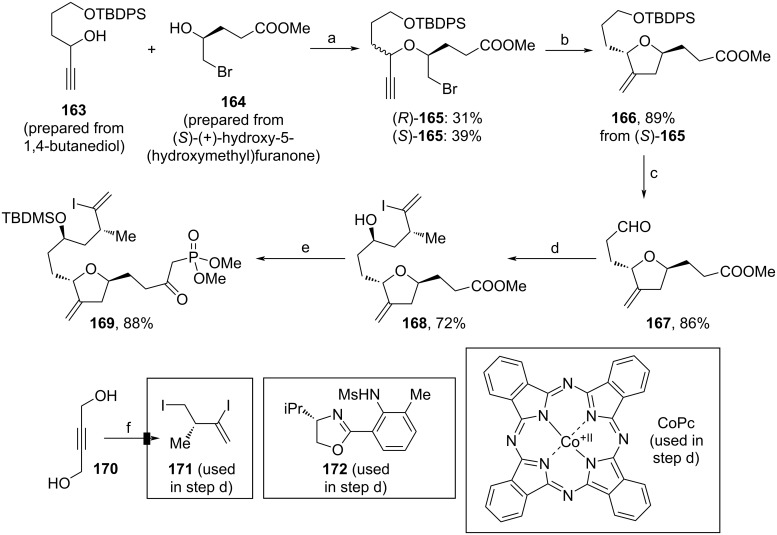
Synthesis of **169**. Reaction conditions: (a) i. Co_2_(CO)_8_, BF_3_·Et_2_O, DCM, 23 °C; ii. CAN, acetone, 0 °C; (b) AIBN, *n*-Bu_3_SnH, toluene, 100 °C; (c) i. TBAF, THF, 0–23 °C; ii. DMP, DCM, 0–23 °C; (d) **171**, CrCl_2_, CoPc, **172**, LiCl, Mn, **39** (proton sponge, see Scheme 5), ZrCp_2_Cl_2_, DME, 23 °C; (e) i. TBDMSOTf, 2,6-lutidine, DCM, −78–0 °C; ii. *N*-BuLi, MePO(OMe)_2_, THF, −78 °C; (f) i. TMSCl, NaI; ii. *m*-MePhCOOH, NaHCO_3_; iii. ClPO(OEt)_2_; iv. AlMe_3_, CuBr; v. *Amano lipase* PS-800; vi. I_2_, PPh_3_, imidazole; CAN: ceric ammonium nitrate.

**Scheme 18:**
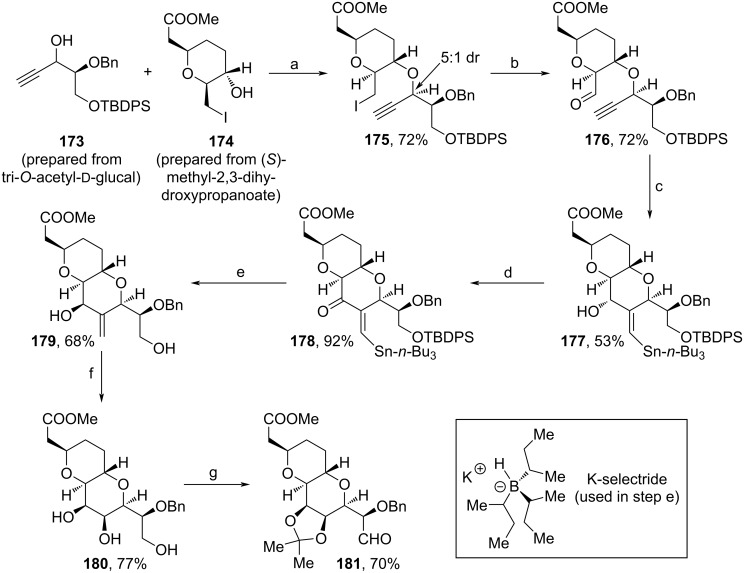
Synthesis of **181**. Reaction conditions: (a) i. Co_2_(CO)_8_, BF_3_·Et_2_O, DCM, 23 °C; ii. (NH_4_)_2_Ce(NO_3_)_6_, acetone, 0–23 °C; (b) DMSO, 2,4,6-collidine, microwave, 180 °C; (c) AIBN, *n*-Bu_3_SnH, toluene, 100 °C; (d) DMP, NaHCO_3_, DCM, 23 °C; (e) i. K-selectride, THF, −20–0 °C; ii. HCl (aq.); (f) i. O_3_, MeOH, −78 °C; ii. NaBH_4_, 23 °C; (g) i. 2,2-dimethoxypropane, *p*-TsOH·H_2_O, acetone, 0–23 °C; ii. DMP, DCM, 0–23 °C.

**Scheme 19:**
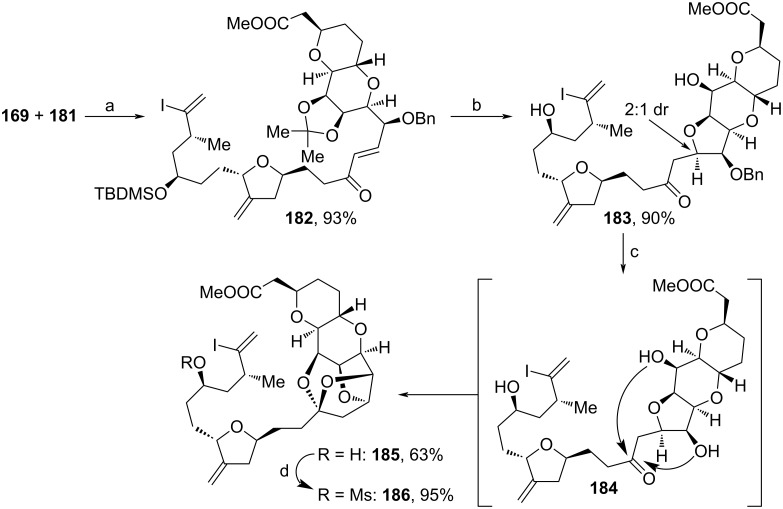
Synthesis of **186**. Reaction conditions: (a) NEt_3_, LiCl, MeCN, 0–23 °C; (b) HF·pyridine, MeCN, 23 °C; (c) DDQ, *h*ν, MeCN, 23 °C; (d) MsCl, NEt_3_, DMAP, DCM, 0 °C.

In the same year, Nicolaou and co-workers achieved the total synthesis of halichondrin B, which included the assembly of C1–C26‘ fragment **186** (Schemes 17–19) [93]. To afford 3-methylene tetrahydrofuran **169**, both starting materials, **163** and **164**, were prepared within few steps from commercially available sources [93]. The etherification of **163** and **164** (Nicholas reaction) led to a diastereomeric mixture, which was separated (Scheme 17). (*S*)-**165** was converted to **166** via radical cyclization, then the TBDPS-protecting group was cleaved and the obtained alcohol oxidized to aldehyde **167**. The Cr(II)/Co(II)-induced asymmetric NHK coupling mediated by **172** with vinyl iodide **171** led to tetrahydrofuran **168**. Protection of the secondary alcohol enabled the conversion to ketophosphonate **169** using MePO(OMe)_2_ and *n*-BuLi. Notably, building block **171** used herein can be synthesized from **170** within 6 steps including a kinetic resolution with *Amano lipase* PS-800. [94]

Another fragment, **181**, was synthesized from alcohols **173** and **174**, which were perpared from tri-*O*-acetyl-ᴅ-glucal and (*S*)-methyl-2,3-dihydroxypropanoate, respectively (Scheme 18) [93]. Again, Nicholas etherification of both starting materials was conducted to afford **175** in 5:1 dr. The major isomer was further transformed to **176** in a microwave oven via Kornblum oxidation. Radical cyclization and DMP-oxidation yielded intermediate **178**, which was reduced with K-selectride, before acidic treatment was applied to remove the TBDPS-protecting group and the tin moiety. From **179**, ozonolysis and subsequent reduction afforded **180** stereoselectively, and eventual protection of the 1,2-diol motif followed by oxidation yielded aldehyde **181**.

Both building blocks from Scheme 17 and Scheme 18 were fused together via HWE reaction to construct **182** (Scheme 19). Treatment with HF·pyridine led to cleavage of the acetonide- and TBDMS-protecting groups, which induced the oxy-Michael cyclization towards **183**. Notably, during this cyclization also considerable amounts of **183**`s epimer were formed (2:1 dr). Oxidation with DDQ removed the Bn-moiety (**184**) and triggered ketalization towards **185**. Eventual mesylation formed key fragment **186** in 95% yield. For the assembly of both heterocyclic subunits of **186**, Nicholas etherification and radical cyclization proved to be suitable, but only moderately yielding, procedures. Further, this pathway, with the exception of the coupling reaction between **167** and **171**, involves only simple and mild transformations using standard reagents. As mentioned above, **186** served as an intermediate for the total synthesis of halichondrin B, which was assembled in a total of 25 steps.

**Scheme 20:**
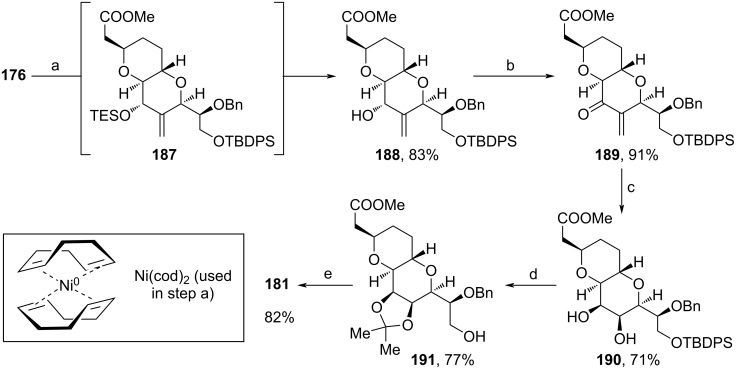
Modified synthesis of **181**. Reaction conditions: (a) i. Ni(cod)_2_, P(*n*-Bu)_3_, Et_3_SiH, THF, 23 °C; ii. HCl (aq), 23 °C; (b) DMP, DCM, 0–23 °C; (c) i. O_3_, MeOH, −78 °C; ii. NaBH_4_, −78–23 °C; (d) HCl (aq), acetone, MeOH, 0–23 °C; (e) DMP, DCM, 0–23 °C.

**Scheme 21:**
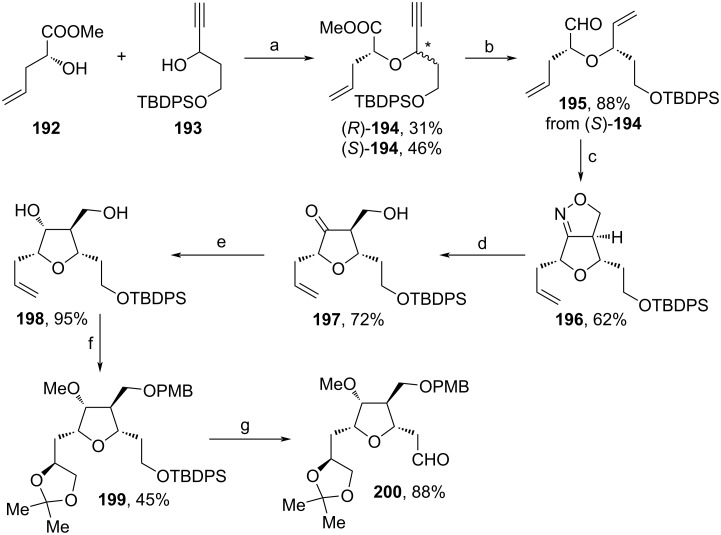
Synthesis of **200**. Reaction conditions: (a) i. Co_2_(CO)_8_, DCM, 23 °C; ii. BF_3_·Et_2_O, 0 °C; iii. (NH_4_)_2_Ce(NO_3_)_6_, acetone; (b) i. Lindlar cat., quinoline, EtOAc, 23 °C; ii. DIBAL-H, DCM, −78 °C; (c) i. NH_2_OH·HCl, pyridine, EtOH, 23 °C; ii. NaOCl, DCM, 23 °C; (d) Mo(CO)_6_, MeCN, H_2_O, 80 °C; (e) Me_4_NBH(OAc)_3_, MeOH, 0 °C; (f) i. PMB-trichloroacetimidate, (±)-CSA, DCM, hexane, 0–23 °C; ii. NaH, MeI, THF, DMF, 0–23 °C; iii. AD-mix α, NH_2_Ms, *t*-BuOH, H_2_O, 5 °C; iv. 2,2-dimethoxypropane, *p*-TsOH·H_2_O, acetone, 23 °C; (g) i. TBAF, THF, 23 °C; ii. DMP, DCM, 0–23 °C.

**Scheme 22:**
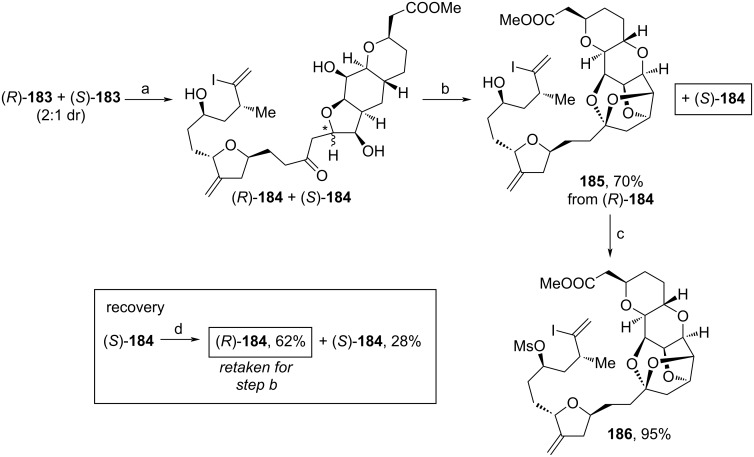
Modified synthesis of **186**. Reaction conditions: (a) DDQ, 2,6-di-*t*-Bu-4-hydroxytoluene, *h*v, MeCN, 23 °C, continuous flow process; (b) *p*-TsOH·H_2_O, 23 °C; (c) MsCl, NEt_3_, DMAP, DCM, 0 °C; (d) NaOMe, MeOH, 23 °C.

**Scheme 23:**
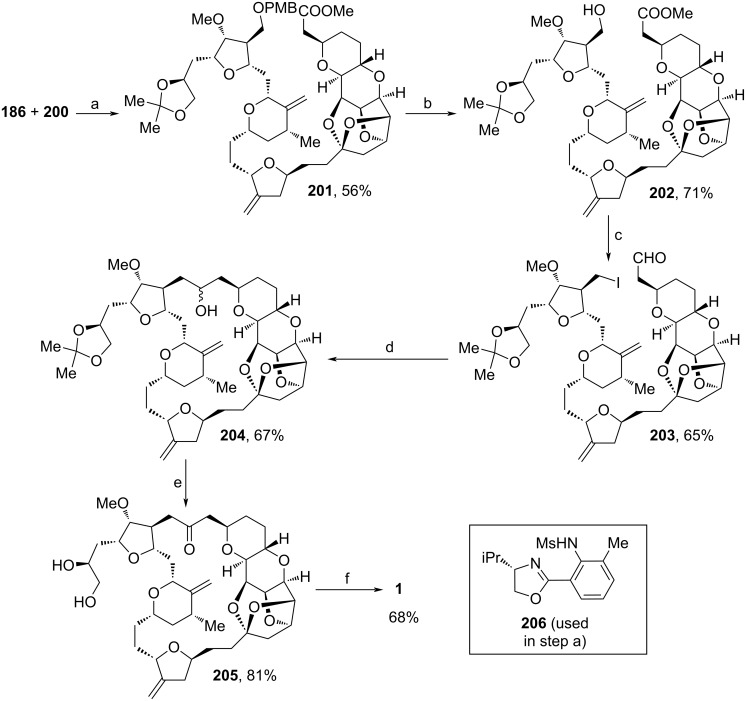
Synthesis of **1**. Reaction conditions: (a) i. CrCl_2_, NiCl_2_, **206**, NEt_3_, THF, 23 °C; ii. DBU, toluene, 110 °C; (b) DDQ, DCM, buffer (pH 7), 23 °C; (c) i. imidazole, PPh_3_, I_2_, THF, 0–23 °C; ii. DIBAL-H, DCM, −78 °C; (d) CoPc (see Scheme 10), KI, CrCl_2_, DMF, 23 °C; (e) i. DMP, DCM, 23 °C; ii. AcOH·H_2_O, 50 °C; (f) i. Ts_2_O, 2,4,6-collidine, pyridine, DCM, −10–0 °C; ii. NH_4_OH, iPrOH, 0–30 °C.

One year later, Nicolaou and co-workers focused specifically on the total synthesis of **1** and therefore optimized few steps from their previous halichondrin B synthesis (Schemes 20–23) [95]. Hence, for the assembly of fragment **181** the route shown in Scheme 18 was intercepted at **176**. Instead of the previous radical cyclization, here, a reductive Ni(II)-induced cyclization afforded **188** (Scheme 20). Oxidation and ozonolysis with subsequent addition of NaBH_4_ yielded diol **190**, whose TBDPS-group was cleaved under acidic conditions, before the 1,2-diol motif was protected as acetonide. Eventually, DMP-oxidation afforded aldehyde **181**.

The synthesis of fragment **200** was accomplished starting from substrates **192** and **193** (Scheme 21). After Nicholas etherification of both substrates, which led to a diastereomeric mixture, (*S*)-**194** was isolated and further treated with Lindlar catalyst and DIBAL-H to afford the alkene and aldehyde motifs of **195**, respectively. Oxime formation and oxidation yielded an intermediate nitrile oxide, which underwent an intramolecular [3 + 2]-cycloaddition with the adjacent ethene substituent towards isoxazoline **196**. Reductive N–O bond cleavage and stereospecific reduction with Me_4_NBH(OAc)_3_ yielded diol **198**, whose primary alcohol unit was PMB-protected, while the secondary one was methylated. Dihydroxylation and protection with 2,2-dimethoxypropane yielded **199**. Then, desilylation with TBAF and DMP-oxidation led to aldehyde **200**.

Another optimization was achieved during the formation of the central ketal motif of **186** (Scheme 22). Here, the diastereomeric mixture of **183** (see Scheme 19) was exposed to light-mediated DDQ-oxidation within a continuous flow setup, which yielded epimers (*R*)-**184** and (*S*)-**184**. This mixture was further treated with *p*-TsOH·H_2_O leading to cyclized **185** from (*R*)-**184** and leftover (*S*)-**184**. While **185** was mesylated within the last step towards **186**, (*S*)-**184** was epimerized upon addition of base, and another fraction of (*R*)-**184** was recovered and also taken for the assembly of **186**.

With **186** and **200** in hand, Nicolaou and co-workers merged both building blocks within a Ni(II)/Cr(II)-catalyzed NHK reaction using chiral ligand **206** (Scheme 23) [95]. Upon treatment with DBU, 3-methylenetetrahydrofuran **201** was obtained in 56% yield over two steps. PMB-deprotection, iodination and selective reduction of the ester moiety with DIBAL-H furnished **203**. The cyclization procedure towards **204** was based on a CoPc/CrCl_2_ coupling protocol by Takai and co-workers, and was optimized to a yield of 67% [96]. DMP-oxidation and acidic cleavage of the acetonide moiety yielded **205** in 81%. Eventual tosylation of the primary alcohol with Ts_2_O and following amination yielded **1**. In comparison to their former route [93], here, the bicyclic motif of **181** was not assembled via tin-mediated radical cyclization reaction, but with Ni-catalyzed cyclization (see Scheme 18 and Scheme 20). This innovation led to a remarkable increasement of overall yield (17.6% vs 9.3%). Also, the ketalization from **183** to **184** was enhanced. Here, the application of a continuous flow process and the recovery of (*R*)-**184** led to an increasement of yield in 6.7% in comparison to the former approach. It is also important to mention that Nicolaou and co-workers paved the way for further derivatives of **1** with this synthesis.

**Scheme 24:**
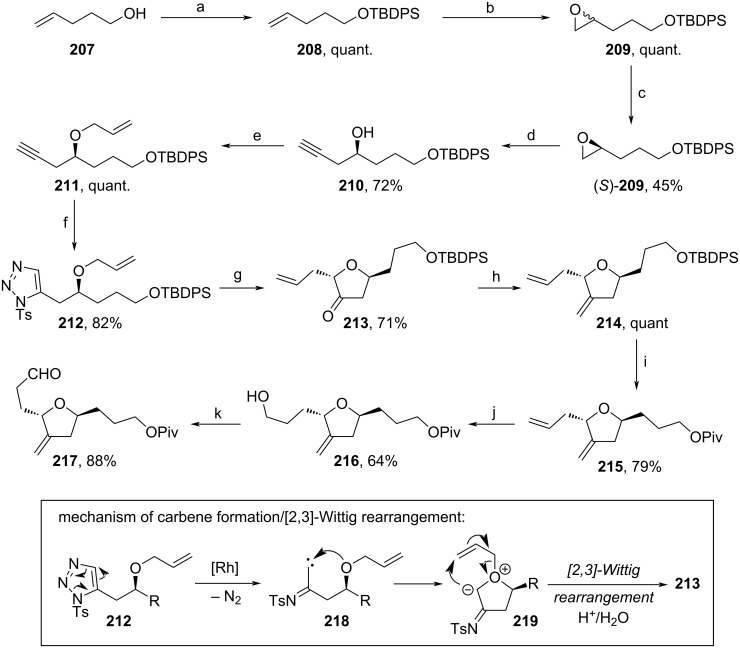
Synthesis of **217**. Above: Reaction conditions: (a) TBDPSCl, imidazole, DCM, 0–5 °C. (b) *m*-CPBA, DCM, 0 °C; (c) Jacobsen cat. (0.5 mol %), H_2_O, rt; (d) ethylenediamine, lithium acetylide, DMSO, 10–15 °C; (e) NaHMDS, allyl bromide, TBAI, DMF, 0 °C; (f) *n*-BuLi, TsN_3_, THF, −60 °C; (g) i. Rh_2_(OAc)_4_ (5 mol %), toluene, 60–90 °C; ii. acidic work-up; (h) MePh_3_PBr, *t-*BuOK, THF; (i) i. TBAF, THF, rt; ii. PivCl, DMAP, DCM, rt; (j) BH_3_·THF, 2,3-dimethyl-2-butene, cyclopentane, −20 °C; (k) DMP, NaHCO_3_, DCM, rt. Below: Mechanism of the [2,3]-Wittig rearrangement in step (g).

A large-scale synthesis of **217** was achieved by Kim and co-workers using a Rh(II)-induced carbene formation and subsequent [2,3]-Wittig rearrangement [97] (Scheme 24). Herein, alcohol **207** was TBDPS-protected, before epoxidation yielded **209**. Hydrolytic kinetic resolution of **209** afforded enantioenriched epoxide (*S*)-**209**, which was opened regioselectively with lithium acetylide. The allylation and [3 + 2]-cycloaddition with tosyl azide led to **212**. Treatment with Rh_2_(OAc)_4_ induced a [2,3]-Wittig rearrangement and subsequent hydrolysis afforded **213** in 71% yield. A detailed mechanism for this transformation is shown in Scheme 24 below [98]. Here, the Rh(II)-induced elimination of N_2_ generates carbene **218**, which is attacked by the adjacent allyl ether moiety to form unstable zwitterion **219**. Sigmatropic rearrangement and acidic workup yielded **213** stereoselectively. Further methylenation of the keto group with MePh_3_PBr and exchange of the alcohol protecting group were used to form **215**. Finally, regioselective hydroboration–oxidation and DMP-oxidation afforded aldehyde **217**. Notably, all reactions were performed in a (multi-)kg scale in consistently high yields, with exception of step (c), showing the applicability of this sequence for production.

**Scheme 25:**
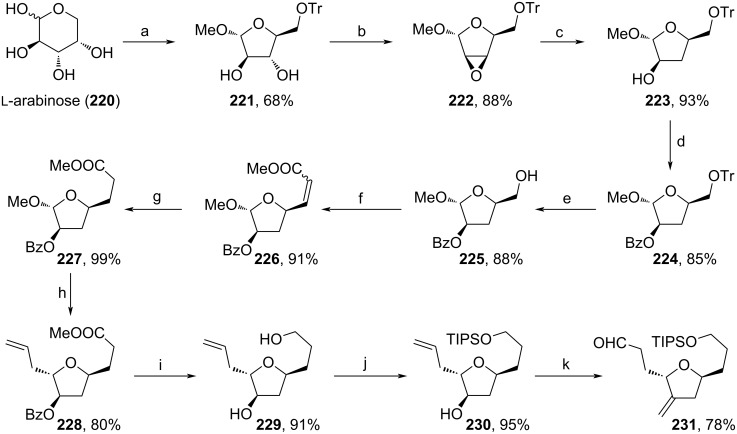
Synthesis of **231**. Reaction conditions: (a) i. AcCl, MeOH, 0 °C to rt; ii. TrCl, pyridine, 50 °C; (b) PPh_3_, DIAD, THF, rt; (c) LiAlH_4_, THF, relfux; (d) BzCl, pyridine, 0 °C to rt; (e) 60% AcOH (aq), 50 °C; (f) i. DMP, DCM, rt; ii. Ph_3_PCHCO_2_Me, toluene, rt; (g) Pd/C, MeOH, H_2_, rt; (h) allyltrimethylsilane, BF_3_·Et_2_O, DCM, 0 °C to rt; (i) LiAlH_4_, THF, rt; (j) TIPSCl, imidazole, DMF, rt; (k) i. DMP, DCM, rt; ii. MePPh_3_Br, *t*-BuOH, THF, rt; iii. Sia_2_BH, THF, −20 °C; iv. DMP, NaHCO_3_, DCM, rt; TrCl: triphenylmethyl chloride.

As a cost-efficient approach towards fragment **231**, Yu and co-workers applied ʟ-arabinose (**220**) as a starting material (Scheme 25) [99]. Herein, **220** was first treated with AcCl in MeOH, then protected with TrCl to yield **221** in 68%. The diol was converted stereospecifically to epoxide **222** under Mitsunobu conditions, then reopened with LiAlH_4_ and protected with BzCl. Deprotection of the primary alcohol was necessary for the consecutive DMP-oxidation towards an intermediate aldehyde and HWE-reaction to assemble **226** as an *E*/*Z* mixture. Hydrogenation of alkene **226** and treatment with allyltrimethylsilane were used to form **228**. The ester moiety of **228** was reduced and the Bz-group was cleaved simultaneously to afford the respective diol (**229**), then TIPS-protection of the primary alcohol unit enabled the oxidation towards an intermediate ketone, which was further methylenated with MePPh_3_Br. Eventually, the regioselective hydroboration–oxidation and DMP-oxidation led to aldehyde **231**. Herein, both stereocenters of **231** were adopted from the naturally given structure of **220** and were maintained over 14 steps with a remarkable total yield of 24%.

**Scheme 26:**
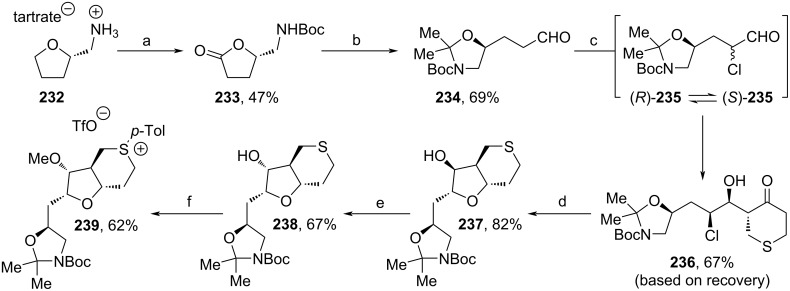
Synthesis of **239**. Reaction conditions: (a) i. Boc_2_O, K_2_CO_3_, THF, rt; ii. Ru(acac)_3_, NaBrO_3_, EtOAc, H_2_O, rt; (b) i. NaBH_4_, MeOH, THF, rt to 60 °C; ii. 2,2-dimethoxypropane, *p*-TsOH, acetone, rt; iii. PCC, celite, DCM, rt; (c) i. ᴅ-proline, NCS, DCM, 0 °C; ii. tetrahydrothiopyran-4-one, DMSO; (d) i. DIBAL-H, THF, −78 °C; ii. SrCO_3_, H_2_O, MeOH, 75 °C; (e) i. *p*-nitrobenzoic acid, DIAD, PPh_3_, THF, 0 °C to rt; ii. NaOH, MeOH, rt; (f) i. NaO*t*-Bu, MeI, THF, 0 °C to rt; ii. *p*-Tol_2_IOTf, Cu(OBz)_2_, DCE, 110 °C.

**Scheme 27:**
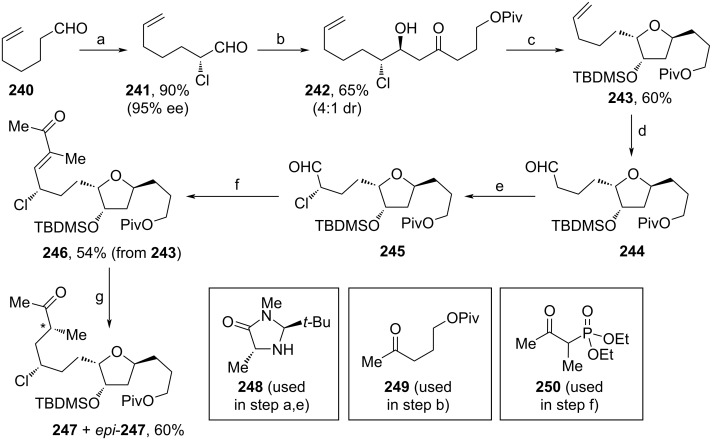
Synthesis of **247**. Reaction conditions: (a) NCS, **248**, MeCN, 0 °C to rt; (b) LDA, **249**, THF, −78 °C; (c) i. DIBAL-H, THF, −78 °C; ii. AgOTf, Ag_2_O, THF, 0 °C to rt; iii. TBDMSOTf, NEt_3_, DCM, 0 °C; (d) OsO_4_, NaIO_4_, THF, H_2_O, rt; (e) NCS, *ent-***248**, MeCN, 0 °C to rt; (f) **250**, Ba(OH)_2_, THF, rt; (g) i. Mn(dpm)_3_, PhSiH_3_, iPrOH, rt; ii. DBU, MeCN, 5 °C; during step g (i) also the epimer of **247** (*epi-***247**) was formed (**247**:*epi-***247**=1.5:1), clean **247** was obtained fractionwise via chromatographical separation of the mixture (**247**+*epi-***247**) after both reaction steps.

**Scheme 28:**
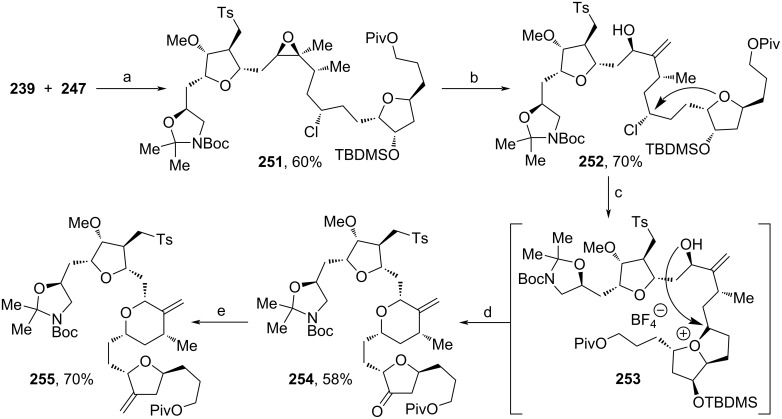
Synthesis of **255**. Reaction conditions: (a) i. LiHMDS, THF, −78 °C to rt; ii. *m*-CPBA, DCM, −78 °C to rt; (b) Cp_2_TiCl_2_, Zn, THF, rt; (c) AgBF_4_, 2,6-di-*t*-Bu-4-methylpyridine, *t-*BuOAc, rt; (d) i. HF·pyridine, THF, rt; ii. DMP, NaHCO_3_, DCM, rt; (e) MePPh_3_Br, *t*-BuOK, THF, 0 °C to rt.

In 2023, Kaghad and co-workers designed a novel route towards C14–C35 fragment **255** involving three stereoselective α-chlorinations of aldehydes as the key steps (Schemes 26–28) [100]. From tartrate salt **232** (obtained from tetrahydrofurfurylamine and ᴅ-(−)-tartaric acid), Boc-protection and Ru(III)-induced oxidation led to lactone **233** (Scheme 26). Treatment with NaBH_4_ triggered the cleavage into an intermediate diol, which was cyclized upon addition of 2,2-dimethoxypropane and oxidized towards aldehyde **234**. The first α-chlorination yielded a racemic mixture ((*R*)-**235** and (*S*)-**235**), which epimerized upon the addition of ᴅ-proline. The subsequent stereoselective aldol reaction with tetrahydrothiopyran-4-one went significantly slower than the epimerization and was directed as well by the amino acid. Therefore, **236** was produced diastereoselectively via dynamic kinetic resolution favoring the (*S*)-enantiomer of **235**. Next, the reduction of the keto moiety of **236** induced a stereospecific cyclization towards **237**, whose stereocenter of the alcohol moiety was inverted under Mitsunobu conditions. Methylation of the newly arranged alcohol group and addition of *p*-Tol_2_IOTf led to the formation of salt **239**.

Additionally, building block **247** was synthesized from commercial **240** (Scheme 27). Again, stereoselective chlorination using NCS and MacMillan catalyst **248** was perfomed to yield **241** [101]. Subsequently, ketone **249** was added to aldehyde **241** within a stereoselective aldol reaction. Reduction with DIBAL-H and addition of AgOTf triggered the cyclization of **242** and the untouched secondary alcohol moiety was TBDMS-protected. **243** underwent dihydroxylation and subsequent oxidative cleavage reaction to furnish **244**, which served as a substrate for the third α-chlorination to afford **245**. HWE-reaction with **250** towards **246** and reduction with PhSiH_3_ led to **247** as a separable mixture of epimers (**247**:*epi-***247** = 1.5:1). Notably, by the treatment of *epi-***247** with DBU, again an epimeric mixture was formed (**247**:*epi-***247** = 1:1) and another fraction of **247** was isolated.

The products from Scheme 26 and Scheme 27 were coupled via Corey–Chaykovsky reaction and the so-obtained epoxide was treated with *m*-CPBA yielding sulfone **251** as the major diastereomer (Scheme 28). Regioselective deprotonation and epoxide opening with in situ-formed Ti(III)-species from Cp_2_TiCl_2_ and zinc led to the formation of **252**. Next, conditions reported by Kishi and co-workers were applied for the nucleophilic substitution of the chloride by the oxygen from the tetrahydrofuran ring towards **253** [94], before an adjacent secondary alcohol group reopened the bicyclic cationic intermediate. Cleavage of the TBDMS-protecting group and DMP-oxidation afforded **254**, which was finally olefinated forming **255**. In this route especially the application of stereoselective α-chlorinations was well demonstrated. Also, the dynamic kinetic resolution towards **236** ressembles a key step of this strategy. With these innovations, **1** can be synthesized formally in 52 steps, which therefore strongly competes with the route from Eisai (67 steps) [66]. Incidentally, by the exchange of starting material **232** (Scheme 26) with ʟ-glutamic acid, Eisai intermediate **12** and an analogous Kishi intermediate were synthesized [19,68].

**Scheme 29:**
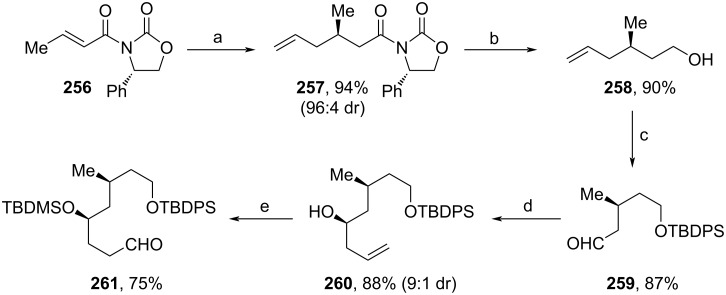
Synthesis of **261**. Reaction conditions: (a) allyltrimethylsilane, TiCl_4_, DCM −78 °C; (b) LiBH_4_, EtOH, Et_2_O, 0 °C; (c) i. TBDPSCl, imidazole, DMAP, THF, 0 °C; ii. O_3_, PPh_3_, DCM, −78 °C; (d) allyltributylstannane, TiCl_4_, Ti(OiPr)_4_, Ag_2_O, (*R*)-BINOL, 0 °C; (e) i. TBDMSOTf, 2,6-lutidine, DCM, 0 °C; ii. BH_3_·SMe_2_, 30% H_2_O_2_, NaOH (aq.), THF; iii. DMP, NaHCO_3_, DCM, 0 °C to rt.

**Scheme 30:**
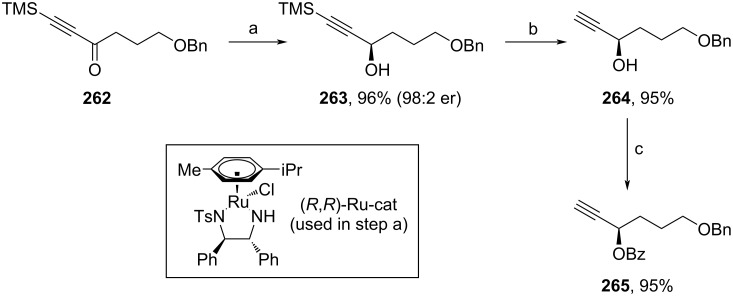
Synthesis of **265**. Reaction conditions: (a) (*R*,*R*)-Ru-cat (0.2 mol %), DCM, NEt_3_, HCOOH, rt; (b) TBAF, THF, 0 °C; (c) BzCl, DMAP, NEt_3_, 0 °C to rt.

**Scheme 31:**
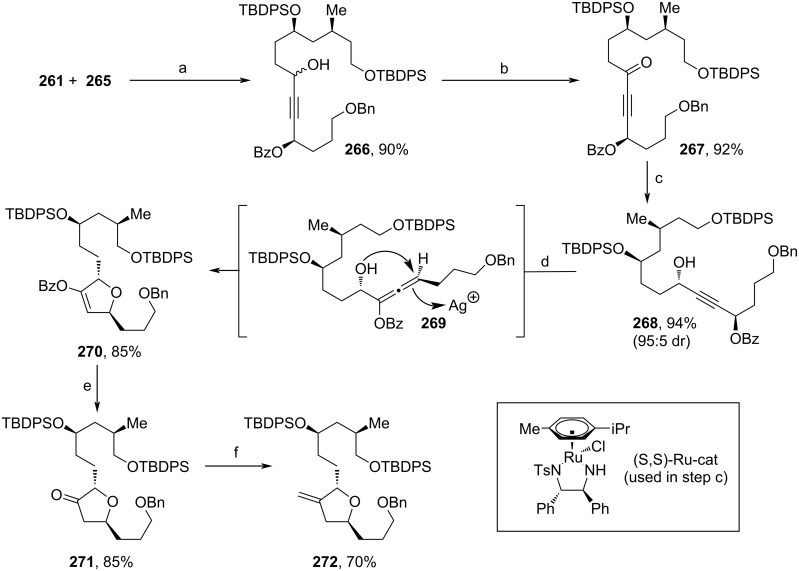
Synthesis of **272**. Reaction conditions: (a) LDA, THF, −78 °C; (b) DMP, NaHCO_3_, DCM, 0 °C to rt; (c) (*S*,*S*)-Ru-cat (0.2 mol %), DCM, NEt_3_, HCOOH, rt; (d) AgBF_4_, cyclohexane, 80 °C; (e) MeLi·LiBr, Et_2_O, −78 °C; (f) MePh_3_PBr, *n*-BuLi, 0 °C.

Shortly thereafter, Nasam and co-workers developed a novel synthesis of **1**`s fragment **272** using multiple asymmetric catalytic transformations (Schemes 29–31) [102]. **256** was synthesized from *trans*-crotonic acid and served as a starting material for the first building block (**261**, Scheme 29). Hosomi–Sakurai allylation of **256** was used to afford **257** [103], which was subsequently treated with LiBH_4_ to yield alcohol **258**. TBDPS-protection and ozonolysis led to aldehyde **259**. The stereoselective allylation was conducted according to a procedure from Maruoka and Kii, and yielded **260** in 9:1 dr [104]. After protection of the secondary alcohol, hydroboration–oxidation of the terminal alkene and treatment with DMP produced aldehyde **261**.

The second building block (**265**) was synthesized via stereoselective Noyori reduction of **262** [105], followed by TMS cleavage and Bz-protection of the free alcohol unit (Scheme 30).

Both products were fused together by the nucleophilic attack of deprotonated alkyne **265** to aldehyde **261** (Scheme 31). The obtained diastereomers **266** were oxidized to the respective ketone **267**, and again, Noyori reduction was performed to access **268** [105]. Treatment with AgBF_4_ at elevated temperature induced a rearrangement via allene **269** and cyclization towards 2,5-dihydrofuran **270**. Cleavage of the Bz-moiety and tautomerization to ketone **271** enabled the unmasking of the methylene motif of **272** via Wittig reaction. In this convergent synthesis, a remarkable overall yield of 17.6% for **272** was obtained, which makes it a competitive reaction sequence for the large-scale process currently applied by Eisai [66]. Furthermore, this method stands out for its cost-efficiency, since only an enantiomeric pair of ruthenium catalysts is needed in addition to other standard chemicals.

**Scheme 32:**
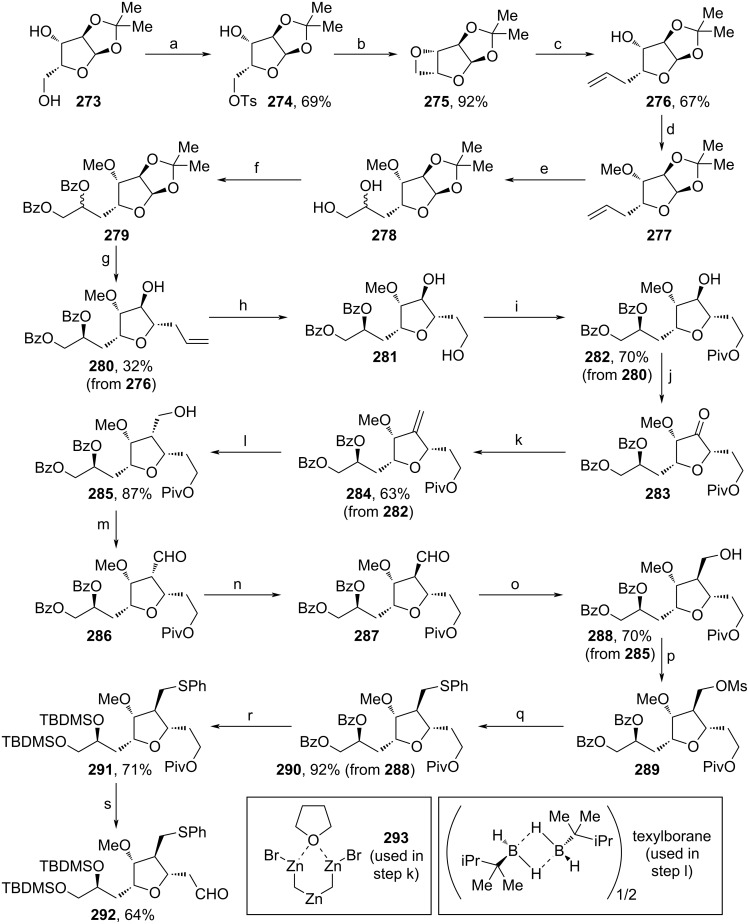
Synthesis of **292**. Reaction conditions: (a) TsCl, NEt_3_, DCM, rt; (b) K_2_CO_3_, MeOH, 45 °C; (c) vinylmagnesium bromide, THF, reflux; (d) NaH, MeI, TBAI, THF, rt; (e) (DHQ)_2_AQN, K_2_OsO_4_·2H_2_O, K_3_Fe(CN)_6_, K_2_CO_3_, *t*-BuOH, H_2_O, 0 °C; (f) *N*-methylmorpholine, BzCl, DMAP, toluene, 75 °C; (g) allylTMS, Ti(OiPr)_4_, TiCl_4_, toluene, rt; (h) i. K_2_OsO_4_·2H_2_O, rt; ii. NaIO_4_, NaHCO_3_, DCM, rt; iii. NaBH_4_, MeOH, rt; (i) PivCl, pyridine, DCM, rt; (j) DMP, NaHCO_3_, DCM, rt; (k) **293**, TiCl_4_, THF, 5 °C to rt; (l) i. texylborane, THF, −20 °C; ii. NaBO_3_·4H_2_O, THF, H_2_O, rt; (m) DMP, NaHCO_3_, rt; (n) NEt_3_, DCM, rt; (o) NaBH_4_, EtOH, 0 °C; (p) Ms_2_O, NEt_3_, DCM, 0 °C; (q) PhSNa, THF, 0 °C; (r) i. Mg(OMe)_2_, MeOH, rt; ii. TBDMSCl, imidazole, DCM, rt; (s) i. DIBAL-H, THF, 0 °C; ii. DMP, NaHCO_3_, rt; (DHQ)_2_AQN: hydrochinin-(anthrachinon-1,4-diyl) diether.

**Scheme 33:**
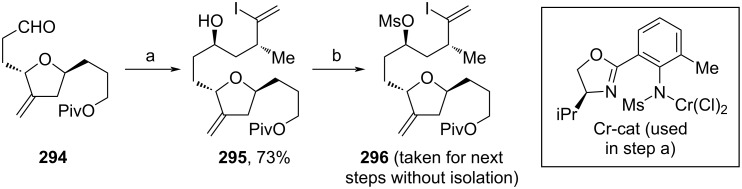
Synthesis of **296**. Reaction conditions: (a) **171** (see Scheme 17), Cr-cat, CoPc (see Scheme 17), Mn, NEt_3_·HCl, LiCl, TMSCl, DME, rt; (b) Ms_2_O, NEt_3_, DCM, −5–0 °C.

**Scheme 34:**
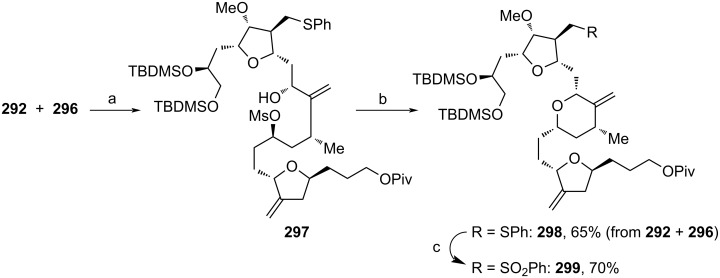
Synthesis of **299**. Reaction conditions: (a) **172** (see Scheme 17), CrCl_2_, NEt_3_, NiCl_2_, THF, rt; (b) KHMDS, THF, −20 °C; (c) (NH_4_)_2_MoO_4_, urea·H_2_O_2_, EtOH, rt.

A similar building block as from Kaghad and co-workers (see Scheme 32) was assembled in a kg-scale by Kim and co-workers intercepting the total synthesis of **1** from Eisai (Schemes 32–34) [100,106]. The synthetic route design could be guided by the patent landscape. This paper is the first to demonstrate that the complex web of legal exclusivities held by an originator company should be a primary consideration when developing a synthetic route for the earliest possible market entry by generic API manufacturer. This strategic approach directly led to the design of non-infringing intermediate **292** for the key NHK coupling with fragment **296** (see Scheme 34). For the assembly of **292**, commercial xylose acetonide (**273**) was Ts-protected, before treatment with K_2_CO_3_ led to formation of oxetane **275** (Scheme 32). Allylation, methylation of the alcohol moiety and dihydroxylation with subsequent Bz-diprotection yielded **279**. After another stereospecific allylation, **280** was isolated as a single diastereomer upon recrystallization. The dihydroxylation, oxidative cleavage and reduction with NaBH_4_ afforded diol **281**, whose primary alcohol unit was protected enabling the selective oxidation towards ketone **283**. Olefination with **293** (Nysted reagent) and stereoselective hydroboration with texylborane and oxidation yielded primary alcohol **285**. Another DMP-oxidation, isomerization under basic conditions, reduction and Ms-protection were conducted to assemble **289**. Substitution of the mesylate with PhSNa, replacement of both Bz- with TBDMS-protecting groups and eventual deprotection of the Piv-moiety, followed by DMP-oxidation led to the formation of the target fragment **292**.

In parallel, **296** was synthesized via Cr(III)-catalyzed coupling of aldehyde **294** with diiodide **171** (see Scheme 17) and subsequent mesylation (Scheme 33).

Stereoselective Cr(II)-catalyzed coupling between aldehyde **292** and iodide **296** with ligand **172** (see Scheme 17) was used for the assembly of **297** (Scheme 34). The addition of KHMDS induced the cyclization towards tetrahydropyran **298**. Finally, oxidation of the sulfide moiety afforded sulfone **299**. Using this synthetic strategy, Kim and co-workers assembled **276** via simple ring opening of **275**`s oxetane moiety and no cooling and intermediate purification were necessary. These practical improvements are particularly important for the large-scale synthesis of **1**. Furthermore, every step up to **292** was carried out in kg amounts using only operationally simple standard procedures.

**Scheme 35:**
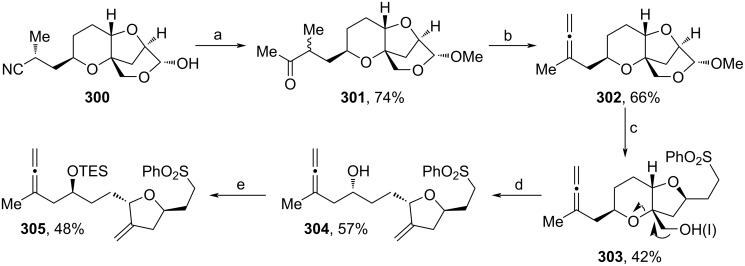
Synthesis of **305**. Reaction conditions: (a) i. *p*-TsOH, MeOH, 40 °C; ii. MeLi, LiBr, THF, −25 °C; (b) i. Tf_2_NPh, NaHMDS, THF, −78 °C; ii. Pd_2_(dba)_3_, (*S*)-MOP, iPr_2_NEt, heptane, 55 °C; (c) i. HCl (aq), THF, rt; ii. PhSO_2_CH_2_PO(OEt)_2_, LiCl, iPr_2_NEt, MeCN, 0 °C to rt; iii. LiBH_4_, THF, 0 °C; (d) i. Tf_2_O, iPr_2_NEt, NaI, DMF, −4 °C to rt; ii. Zn, AcOH, THF, 0 °C; (e) i. *p-*nitrobenzoic acid, PPh_3_, DIAD, toluene, 0 °C; ii. LiOH·H_2_O, THF, H_2_O, MeOH, rt; iii. TESCl, imidazole, DCM, 0 °C to rt; (*S*)-MOP: (*S*)-2-diphenyphosphino-2'-methoxyl-1,1'-binaphthyl.

**Scheme 36:**
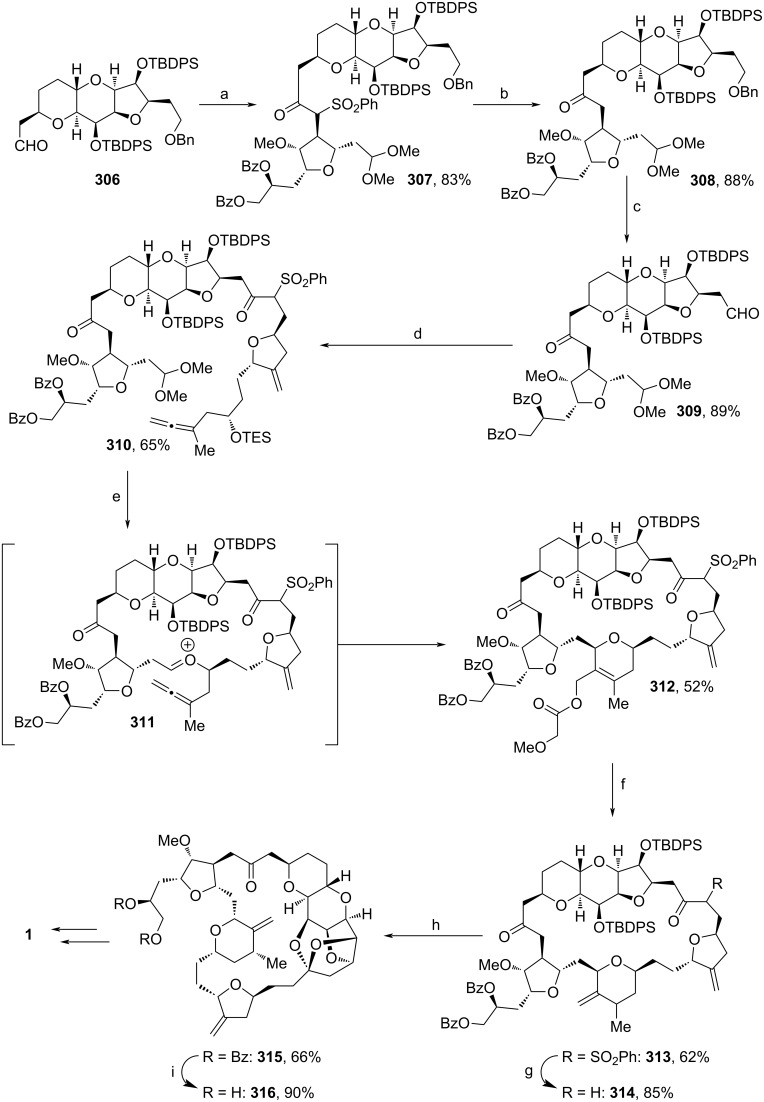
Synthesis of **1**. Reaction conditions: (a) i. **41** (see Scheme 6), LDA, THF, −78 °C; ii. DMP, NaHCO_3_, DCM, rt; (b) SmI_2_, THF, MeOH, −78 °C; (c) i, Pd/C, H_2_, EtOAc, MeOH, rt; ii. DMP, NaHCO_3_, DCM, rt; (d) i. **305**, *n*-BuLi, *t*-BuOK, THF, −78 °C; ii. DMP, NaHCO_3_, pyridine, DCM, rt; (e) methoxyacetic acid, BF_3_·OEt_2_, DCM, –25 to −10 °C; (f) Pd(PPh_3_)_4_, PPh_3_, HCOOH, NEt_3_, THF, 60–65 °C; (g) SmI_2_, THF, MeOH, −78 °C; (h) i. TBAF, imidazole·HCl, *N*,*N*-dimethylazetimide, THF, rt; ii. pyridinium *p*-toluenesulfonate, DCM, rt; (i) Mg(OMe)_2_, MeOH, rt.

Employing their recent protocol on allene-Prins reactions (see Scheme 6), Kim and co-workers developed a completed route towards **1**, which included no chromium reagents (Scheme 35 and Scheme 36) [107]. Therefore, **300** was synthesized from ᴅ-quinic acid according to a known procedure [79]. Acidic treatment in MeOH and subsequent addition of MeLi furnished full acetal **301** (Scheme 35). Treatment with Tf_2_NPh yielded an intermediate enol triflate, which underwent a β-hydride elimination towards allene **302** upon addition of Pd_2_(dba)_3_. Next, ring opening of the full acetal to obtain the respective aldehyde and alcohol motifs were accomplished by acidic treatment, then HWE reaction and 1,4-reduction with LiBH_4_ afforded sulfone **303**. The iodination and addition of Zn in AcOH triggered a Bernet–Vasella-type fragmentation (indicated by arrows) towards **304** [108]. Finally, Mitsunobu inversion of the secondary alcohol and TES-protection yielded allene **305** in 48%.

The assembly of all building blocks is shown in Scheme 36. **306** was derived from the TBDPS-protection and DIBAL-H reduction of **92** (see Scheme 9). Deprotonated **41** (see Scheme 6) was added to **306**`s aldehyde motif, before DMP-oxidation yielded ketone **307**. SmI_2_-induced defunctionalization of the sulfone unit afforded **308** in 88%. Afterwards, the Bn-moiety was cleaved off and the compound was oxidized to the respective aldehyde **309**, which enabled the reaction with building block **305** towards **310** via interrupted Julia coupling. The Prins-cyclization via **311** proceeded upon addition of BF_3_·OEt_2_ and methoxyacetic acid. From **312**, Tsuji-reduction and, again, defunctionalization with SmI_2_ led to the formation of **314**. Desilylation followed by ketalization under acidic conditions furnished diol **316**. The final transformation towards **1** is known from previous works and can be accomplished by regioselective tosylation of **316**`s primary alcohol unit and subsequent amination [66]. Notably, in this sequence, 3 fragments are synthesized individually (**41**, **305** and **306**) and finally merged in a short sequence (**306→310**) to the target macrocycle. Hence, this pathway enables the quick assembly of multiple derivatives. The late-stage allene-Prins reaction also facilitates the application of other substituents on the central 3-methylenetetrahydropyran unit. On top of that, the commonly used NHK reaction involving a Cr(II)-catalyst is bypassed [66], which makes the pathway cheaper and eco-friendlier.

**Scheme 37:**
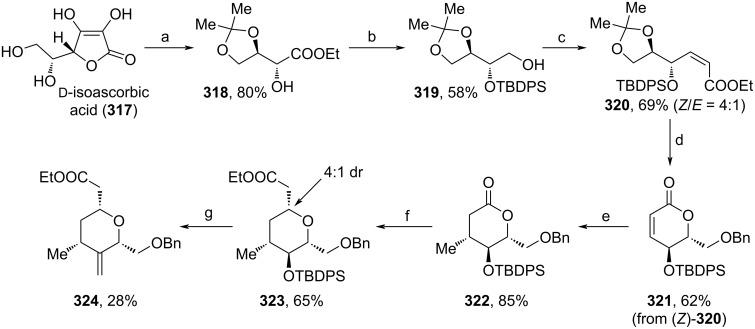
Synthesis of **324**. Reaction conditions: (a) i. acetone, CuSO_4_, rt; ii. H_2_O_2_ (30%), K_2_CO_3_, rt; iii. EtI, MeCN, 70 °C; (b) i. TBDPSCl, DCM, rt; ii. LiBH_4_, THF, rt; (c) i. 2-iodoxybenzoic acid, MeCN, 90 °C; ii. NaH, (PhO)_2_POCH_2_COOEt, THF, −78 °C; (d) i. *p*-TsOH·H_2_O, benzene, rt; ii. Ag_2_O, BnBr, toluene, rt; (e) Me_2_CuLi, TMSCl, Et_2_O, −20 °C; (f) i. DIBAL-H, DCM, −78 °C; ii. triethyl phosphonoacetate, NaH, THF, rt; (g) i. TBAF, THF, rt; ii. DMP, DCM, rt; iii. methyltriphenylphosphonium bromide, *n*-BuLi, THF, 0 °C.

Very recently, Khatravath and co-workers used ᴅ-isoascorbic acid (**317**) as a starting material for the synthesis of tetrahydropyran fragment **324** (Scheme 37) [109]. First, **317** was protected as acetonide, before oxidative cleavage and esterification with EtI were conducted to form **318**. TBDPS-protection and reduction yielded alcohol **319** in 58%, which was further oxidized to the respective aldehyde and olefinated leading to a 4:1 (*Z*/*E*) mixture of **320**. The isomers were separated via chromatography, then, (*Z*)-**320** was deprotected under acidic conditions, which led to a cyclization, and benzylated with BnBr towards **321**. Stereoselective Michael addition of a Gilman reagent yielded **322**. Reduction of the lactone unit yielded a lactol, which is in equilibrium with acyclic aldehyde and alcohol. The aldehyde was trapped via HWE reaction and the obtained α,β-unsaturated carbonyl was reattacked by the alcohol (oxy-Michael reaction, see reaction sequence in Scheme 10) leading to a diastereomeric mixture of **323** (4:1 dr). **323** was deprotected, oxidized and methylenated to afford **324** in 28% yield. Overall, **324** was obtained in a total yield of 3% over a sequence of 12 steps, which only included the use of cheap materials and preparatively simple transformations. Therefore, if the overall yield can be increased even further, this synthesis represents a useful alternative to existing pathways.

## Conclusion

In summary, numerous advancements were made in the field of the synthesis of **1**. This is especially observable for the synthesis of the 4 heterocyclic key fragments or intermediates obtained during the Eisai process. Here, the major progresses can be registered in terms of enhancing the sustainability of pathways through the removal of metal catalysts, use of cheap and readily available substrates and reagents, application of mild reaction conditions, and improving step economy and scalability of the process. Moreover, 3 new completed total syntheses were developed by Lee, Nicolaou and Kim, which show improvements in terms of overall yield or enable the targeted derivatization of **1** for future works [110]. An overview of methods, including the respective fragments, steps and scales, is shown in Table 1. Given the importance of **1** for medicine and the reasearch interest in **1**`s derivatives to potentially enhance its potency, this research field will undoubtably continue to grow in the following years.

**Table 1:** Summary and comparison of methods.

Authors, Date	Fragment of **1**	No. of steps^a^	Scales^b^	References

Konda, 2016	C27–C35	16 and 12	mg–g	[72]
Lee, 2016	total synthesis	71 (LLS^c^: 33)	mg	[73]
Choi, 2017	C14–C35	51 (LLS: 29)	mg–g	[78]
Gaddam, 2018	C1–C13 with C28–C35	27	mg–g	[80]
Kim, 2018	C1–C15	29 (from **66**)	mg–g	[82]
Kim, 2019	C1–C14	15	g	[84]
Lee, 2019	C1–C13	23	g–kg	[86]
Kathravath, 2019	C1–C11	17	mg–g	[87]
Krishna, 2020	C14–C19‘	5	g	[88]
Mallurwar, 2021	C14–C21	16 and 15	mg–g	[89]
Kim, 2021	C1–C13	21	kg	[90]
Senapati, 2021	C14–C28	16	mg–g	[91]
Nicolaou, 2021	C1–C26‘	35 (LLS: 17)	mg–g	[93]
Nicolaou, 2022	total synthesis	62 (LLS:28)	mg–g	[95]
Kim, 2022	C14–C23	13	kg	[97]
Yu, 2023	C14–C23	16	g	[99]
Kaghad, 2023	C14–C35	30 (LLS: 20)	mg–g	[100]
Nasam, 2023	C14–C26	17 (LLS: 14)	mg–g	[102]
Kim, 2024	C14–C35	29 (LLS: 27)	g–kg	[106]
Kim, 2025	total synthesis	74 (LLS: 32)	mg–g	[107]
Kathravath, 2025	C22–C28	15	mg–g	[109]

^a^Starting from commercial sources; ^b^Scales of reactions; ^c^Longest linear sequence.

## Data Availability

Data sharing is not applicable as no new data was generated or analyzed in this study.
